# Docosahexaenoic Acid for Reading, Cognition and Behavior in Children Aged 7–9 Years: A Randomized, Controlled Trial (The DOLAB Study)

**DOI:** 10.1371/journal.pone.0043909

**Published:** 2012-09-06

**Authors:** Alexandra J. Richardson, Jennifer R. Burton, Richard P. Sewell, Thees F. Spreckelsen, Paul Montgomery

**Affiliations:** Centre for Evidence-Based Intervention, University of Oxford, Oxford, United Kingdom; The University of Queensland, Australia

## Abstract

**Background:**

Omega-3 fatty acids are dietary essentials, and the current low intakes in most modern developed countries are believed to contribute to a wide variety of physical and mental health problems. Evidence from clinical trials indicates that dietary supplementation with long-chain omega-3 may improve child behavior and learning, although most previous trials have involved children with neurodevelopmental disorders such as attention-deficit/hyperactivity disorder (ADHD) or developmental coordination disorder (DCD). Here we investigated whether such benefits might extend to the general child population.

**Objectives:**

To determine the effects of dietary supplementation with the long-chain omega-3 docosahexaenoic acid (DHA) on the reading, working memory, and behavior of healthy schoolchildren.

**Design:**

Parallel group, fixed-dose, randomized, double-blind, placebo-controlled trial (RCT).

**Setting:**

Mainstream primary schools in Oxfordshire, UK (n = 74).

**Participants:**

Healthy children aged 7–9 years initially underperforming in reading (≤33^rd^ centile). 1376 invited, 362 met study criteria.

**Intervention:**

600 mg/day DHA (from algal oil), or taste/color matched corn/soybean oil placebo.

**Main Outcome Measures:**

Age-standardized measures of reading, working memory, and parent- and teacher-rated behavior.

**Results:**

ITT analyses showed no effect of DHA on reading in the full sample, but significant effects in the pre-planned subgroup of 224 children whose initial reading performance was ≤20^th^ centile (the target population in our original study design). Parent-rated behavior problems (ADHD-type symptoms) were significantly reduced by active treatment, but little or no effects were seen for either teacher-rated behaviour or working memory.

**Conclusions:**

DHA supplementation appears to offer a safe and effective way to improve reading and behavior in healthy but underperforming children from mainstream schools. Replication studies are clearly warranted, as such children are known to be at risk of low educational and occupational outcomes in later life.

**Trial Registration:**

ClinicalTrials.gov NCT01066182 and Controlled-Trials.com ISRCTN99771026

## Introduction

Omega-3 fatty acids are dietary essentials, but intakes are low by historical standards in most modern developed countries [1]. The longer-chain omega-3 found in fish, seafood and some algae – known as docosahexaenoic acid (DHA) and eicosapentaenoic acid (EPA) – are the most biologically important forms, not only for cardiovascular and immune system health, but also for normal development and functioning of the brain and nervous system [2]. Accumulating evidence from epidemiological, biochemical and intervention studies suggests that low dietary intakes of these long-chain omega-3 may have a detrimental effect on children's behavior and cognitive development [3], [4].

Prior to this study there was already some evidence from randomized, controlled trials (RCTs) that dietary supplementation with omega-3 long-chain, polyunsaturated fatty acids (LC-PUFAs) may have benefits for child behavior and learning [5], [6]. However, almost all such studies had involved populations with specific developmental conditions such as attention-deficit/hyperactivity disorder (ADHD), dyslexia or developmental coordination disorder (DCD). They were also small trials with considerable differences between the populations studied, treatment formulations used, and outcomes assessed. Findings from these RCTs had therefore been mixed, but the most consistently reported benefits in children of school age had included improvements in attention and concentration, and reductions in other ‘ADHD-type’ symptoms such as impulsive and oppositional behaviour, as well as anxiety and emotional lability [5], [6]. Highly significant improvements in both reading and spelling performance were also found in the one study that assessed these outcomes [7].

These findings raised the important question of whether such results might have broader applicability. A systematic review of the effects of omega-3 intake on child behavior and learning, carried out in 2006 for the UK Food Standards Agency, emphasized that findings from groups of children with varying levels of clinically reported neurodevelopmental disorder could not reliably be used to assess the potential effects of omega-3 fatty acids on the educational performance of mainstream UK school children [8]. There was thus a clear need for RCTs involving healthy children from the general school population. In designing such a study, however, we reasoned that any benefits from omega-3 supplementation would more likely be demonstrable in children who were initially underperforming on the outcomes of interest. Official figures show that 20% of all children in mainstream UK schools are in need of additional learning support [9]. Given the importance of literacy skills to children's educational progress, reading achievement was chosen as a primary outcome in this study; and we decided to focus simply on those children whose current reading performance placed them within the bottom 20% of the general population distribution.

Treatment formulations were another important issue. Most previous research had used varying mixtures of EPA and DHA (the two main omega-3 LC-PUFAs found in fish oils) and sometimes other ingredients, making it difficult to identify which component(s) might be responsible for any treatment effects. DHA is an essential structural component of neuronal membranes, and therefore the main omega-3 found in brain and nerve tissue. Furthermore, the ability of humans to synthesize DHA in-vivo from shorter-chain, plant-derived omega-3 such as alpha-linolenic acid, is very limited, making a direct dietary supply of DHA particularly important [2].

This study was therefore designed to investigate the importance of DHA for behavior and learning in healthy but underperforming children from the mainstream school population. The study outcomes were selected for their relevance to children's educational progress and future life chances, and involved simple, practical measures of reading, working memory and behavior.

(a) *Reading*: Literacy skills are fundamental to educational and occupational success. Only 85 per cent of UK adults had a basic level of functional literacy in 2005, and in 2006, five million adults were judged to be under-functioning in this domain [10]. These statistics are an issue of major public concern; and given the dynamic and cumulative nature of the development of literacy skills in children, early intervention is known to be more cost-effective than later remediation [11].

(b) *Working memory*: The ability to hold and manipulate information in the short-term is important for many aspects of everyday life as well as educational performance; and furthermore, working memory problems (particularly the accurate processing and retrieval of auditory/verbal sequential information), are commonly associated with reading difficulties [12], [13]. (c) *Behavior* : Difficulties with behavior in childhood are one of the best predictors of poor educational and occupational achievements in later life. Such problems affect an increasing proportion of UK schoolchildren, and again early intervention is key to minimising the adverse consequences to individuals, families and wider society [14].

### Objectives

To investigate the effects of dietary supplementation with 600 mg/day of the omega-3 LC-PUFA docosahexaenoic acid (DHA) on children's reading, working memory and behavior over a 16-week period.

The hypothesis was that DHA would be of benefit in each of these domains.

## Methods

The protocol for this trial and supporting CONSORT checklist are available as supporting information; see Protocol S1 and Checklist S1.

This was a parallel group, fixed-dose, randomized, double-blind, placebo-controlled trial (RCT).

### Participants

The study was open to healthy children attending any mainstream Oxfordshire primary school who were in the third, fourth or fifth year-groups. Such children are typically aged 7–9 years, although a minority is aged 6 or 10 years.

As originally designed, the inclusion criteria required children to be below the 20th centile on a age-standardized word reading test normed on UK children [15]. In children within this age range, this would typically equate to a reading performance of around 2 years below the level expected for the child's chronological age. Before first randomization, however, it became apparent that planned study numbers would not be achieved unless this inclusion criterion was relaxed to the 33^rd^ centile (equivalent to reading at around 18 months behind chronological age); hence, the protocol was modified accordingly.

Children with specific medical disorders (e.g. visual or hearing impairment), general learning difficulties, or who were taking medications expected to affect behavior and learning, were excluded from the study, as were those whose first language at home was not English. Schools were also asked to exclude any children whose social/family circumstances would have made inclusion into the study inappropriate (e.g. serious illness in the family). Children who, according to their parents, ate fish more than twice a week or took omega-3 supplements were also excluded.

**Figure 1:**
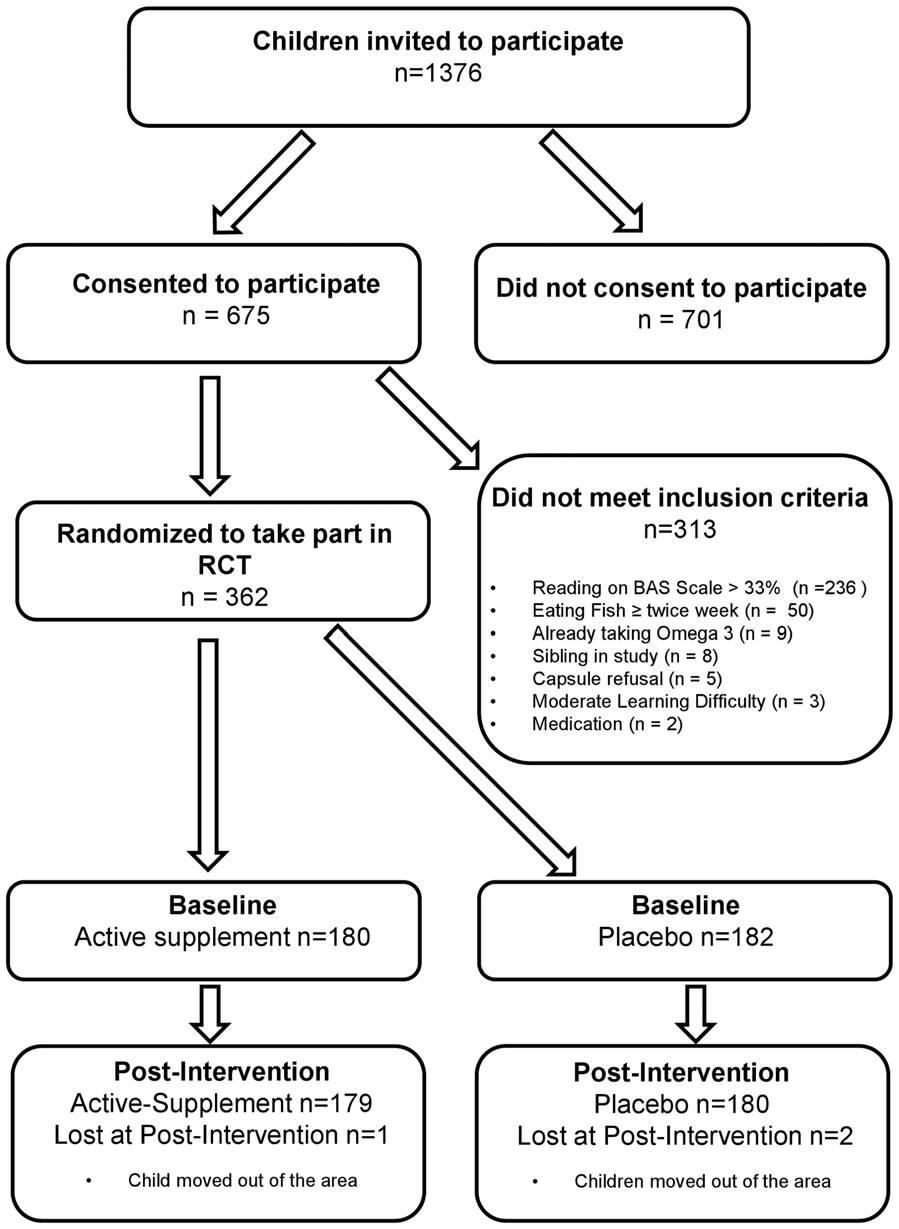
Flow of participants from invitation to randomization.

The Oxfordshire Local Authority was an active partner in the research, and they provided information on how children from participating schools had performed on the national attainment tests conducted on all 7 year olds in state schools in England, Wales and Northern Ireland [16]. From this, an initial list was drawn up of all children with below average attainment in reading at that age. Teachers at participating schools were then invited to modify this according to their opinions of the children's *current* reading performance. On this basis, letters were sent to parents inviting their children to take part in the formal screening assessments, in which their reading ability was individually tested to determine eligibility (see Figure 1).

### Ethics

Written informed consent was gained from parents, and verbal assent from the children, prior to the initial screening assessments. The protocol for this trial and a CONSORT checklist are included as supporting information. Ethical consent was gained from the Milton Keynes Research Ethics Committee (08/H0603/49).

### Interventions

Children in the Active group received a fixed dose of 600 mg DHA (from algal oil), delivered in three 500 mg capsules per day, each providing 200 mg DHA. The Placebo treatment consisted of three 500 mg capsules per day containing corn/soybean oil, matched with the Active treatment for taste and color. Both treatments were provided by Martek Biosciences Corporation (now DSM Nutritional Lipids).

Schools were given a 16 week supply of capsules (labelled with each participating child's name) and asked to dispense 3 capsules to all participating children once a day at lunch time during school terms. Parents were also given a 16-week supply of capsules and asked to dispense these to their children at weekends, during school holidays and at any other time when their children were not in school. Both schools and parents were given full instructions for dispensing capsules and a diary to record capsule consumption, aimed at both monitoring and encouraging children's compliance. Both schools and parents were encouraged to telephone the research team at any time if they had any concerns.

### Outcomes

Primary outcomes, assessed at the baseline screening appointment for all children, and again at 16-week follow-up for the children randomised, were as follows:

- **Reading.** This was assessed using the Word Reading Achievement sub-test of the British Ability Scales (BAS II) [15]. This is a widely used age-standardized, single word reading test, normed on UK children, and sensitive enough to show significant change over four months. Standardized scores have a mean of 100 and a standard deviation of 15.
- **Working memory.** This was assessed via two further sub-tests from the British Ability Scales: Recall of Digits Forward and Recall of Digits Backward. Again, these measures are age standardized, but use T-scores, which have a mean of 50 and a standard deviation of 10.
- **Behavior.** This was assessed by both teachers and parents using the long versions of the Conners' Rating Scales (CTRS-L and CPRS-L) [17]. These are age-standardized, highly valid and reliable scales, measuring child behavior over several domains, expressed as T-scores (mean = 50, sd = 10). They are symptom scales, thus for values above the mean, higher scores indicate more severe difficulties with behaviour and/or attention. For many years these scales have been routinely used in medication trials for children with behavior problems such as ADHD; and they have also been used with success in several previous trials of omega-3 fatty acid supplementation.

### Other measures

#### i) Demographic information

Information on eligibility for free school meals (FSM) was provided by Local Authority data and used as a proxy for Social Economic Status (SES) [18]. Local Authority data were also used to report on participant ethnicity, gender and age.

#### ii) Medication

Details were collected from parents/guardians on current use of medication.

#### iii) Compliance

Compliance was assessed by counting the capsules returned.

#### iv) Side effects

Side effects were recorded using the Barkley Side Effects Rating Scale, a commonly-used instrument assessing the frequency and severity of 17 common side effects which may occur as the result of taking medication or supplements. Each symptom is rated on a 10-point scale from absent to severe [19].

### Description of Procedures

#### Baseline

Baseline assessments took place in schools during normal school hours in a quiet room by two trained researchers. Each child was assessed individually on reading and working memory, and behavior questionnaires were given to the teachers to complete. Parents were sent questionnaires by post.

#### Post-intervention

Children were re-assessed at school 16 weeks post-intervention, when all primary outcome measures were repeated. Upon completion of the study, all participants were given a three months' supply of the Active supplement, as well as a £5 gift token.

### Sample size

Power calculations were performed based on the only previous RCT to assess both reading ability and behavior in children [7]. The results indicated that 180 participants per group would provide 90% power with an α of 5%, for an effect size of r = −0.169 (Cohen's d = −0.343).

### Randomization

A statistician at the Centre for Statistics in Medicine in Oxford independently performed the randomization. The program included a minimization algorithm to ensure balanced allocation of participants across the treatment groups for school (to control for possible between-school differences) and sex (a potentially important factor) [20]. Randomisation was performed only after eligibility was assured, and it was concealed until after the initial two-group analyses were complete.

### Blinding

Investigators, participants and those assessing outcomes were all blind to treatment allocation. Post-intervention, both teachers and parents of participants were asked whether they thought their child had been allocated to Active treatment or Placebo, and these estimates were used to assess the maintenance of blinding.

### Statistical methods

Group comparisons were carried out on primary outcomes using change scores (i.e. the post-intervention score minus baseline score), in line with previous studies. These were considered likely to be more sensitive to treatment effects than total scores, given the short intervention period of 16 weeks. Main analyses were conducted on an intention-to-treat principle (ITT): thus, all children were included according to treatment allocation, irrespective of compliance with the intervention. Appropriate checks were made that participants with missing data did not differ significantly on any demographic variables. Missing data were imputed using median values by treatment group. For all primary outcomes, additional planned group comparisons were carried out on the subgroup of children whose baseline reading scores were ≤20^th^ centile (based on the original protocol), and also on those ≤10^th^ centile (to evaluate any possible trends related to the severity of initial reading problems).

As recommended for the credibility of subgroup analyses [21], tests of interaction were also carried out to assess potential differences in treatment efficacy between the two main subgroups, i.e. all children with initial reading ≤20^th^ centile and those children with milder degrees of initial reading impairment.

For any measures with more than 15% missing data, additional per-protocol analyses were conducted i.e. including only those participants with complete data.

## Results

### Recruitment

Recruitment was carried out in 74 Oxfordshire mainstream primary schools beginning in January 2009 and finishing in November 2010. Post-intervention assessments (16 weeks after enrolment) were completed in April 2011. Of the 1376 children who were invited, 675 of their parents/guardians gave consent and their children were assessed. Of these, 362 met study inclusion criteria and were randomized. The most common reasons for exclusion were that their reading exceeded the 33^rd^ centile (n = 236), followed by eating fish more than twice a week (n = 50); other reasons for exclusion are described in the flowchart of participants (n = 27) detailed in Figure 1.

### Follow-up

Of the 362 children randomized, 359 were assessed again after the 16-week intervention (179 Active, 180 Placebo). Three children were lost at follow up (1 in the Active group, 2 in the Placebo group). In addition, one child from the Placebo group failed to complete the Recall of Digits assessment at the post-intervention follow-up.

### Baseline data

The two treatment groups did not differ on any of the core demographic variables, nor on any of the primary outcome measures at baseline. Demographic information is provided in Table 1. The mean age of the sample was 8 years 8 months, 53% were male, 91% were white, and just over 20% were eligible for free school meals (our proxy for low socio-economic status). Baseline data on the primary outcomes are shown in Table 2. With respect to these, mean reading performance of the children randomized was 1.5 sd below normal, equating to a reading performance around 18 months below chronological age. Working memory scores were around 0.5 to 1 sd below population norms as derived from the British Ability Scales II [15]. On the behavior measures (where higher scores indicate greater difficulties), both teacher and parent ratings were all within the normal range, with the exception of the ‘cognitive problems’ sub-scale (assessing attentional and related difficulties), where these children scored 1 sd above population means. A few other scales showed slight elevations (>+0.5 sd), including ‘oppositional’ (both parents and teachers), ‘anxious-shy’ (teacher ratings only) and both ‘social problems’ and ‘psychosomatic’ (parent ratings only).

**Table 1:** Demographic Information.

	Whole group (n = 362)	Active (n = 180)	Placebo (n = 182)
**Age in months, mean (sd)**	104.3 (10.1)	103.7 (10.0)	104.8 (10.1)
**Sex, n (%)**			
**Male**	192 (53.0)	96 (53.3)	96 (52.7)
**Female**	170 (47.0)	84 (46.7)	86 (47.3)
**Ethnicity, n (%)**			
**White**	330 (91.2)	163 (90.6)	167 (91.8)
**Mixed**	16 (4.4)	8 (4.4)	8 (4.4)
**Other**	7 (1.9)	3 (1.7)	4 (2.2)
**Asian**	2 (0.6)	2 (1.1)	0 (0.0)
**Black**	1 (0.3)	0 (0.0)	1 (0.5)
**Unknown**	6 (1.7)	4 (2.2)	2 (1.1)
**Eligibility for free school meals, n (%)**			
**Not eligible**	289 (79.8)	144 (80.0)	145 (79.7)
**Eligible**	73 (20.2)	36 (20.0)	37 (20.3)

**Table 2:** Primary Outcomes at Baseline, means (sd).

	Whole sample (n = 362)	Active (n = 180)	Placebo (n = 182)
**READING** *			
**Word Reading – Standard Score (sd)** §	84.7 (6.3)	84.6 (6.6)	84.8 (6.7)
***Reading age, months (sd)***	*86.6 (9.9)*	*86.1 (10.0)*	*87.1 (9.7)*
**WORKING MEMORY**			
**Digits Forward – T-Scores** † **(sd)**	40.5 (8.0)	41.0 (8.2)	40.0(7.7)
**Digits Backward – T-scores** † **(sd)**	44.0 (6.8)	44.0 (6.4)	44.0 (7.1)
**BEHAVIOR**			

Teacher rated‡ Sub-scales (T-scores)†	Whole sample (n = 329)	Active (n = 158)	Placebo (n = 171)
**Oppositional**	55.5 (13.4)	54.5 (12.6)	56.3 (14.2)
**Cognitive Problems**	61.6 (9.3)	61.8 (9.8)	61.5 (8.9)
**Hyperactivity**	53.4 (10.9)	53.4 (11.0)	53.4 (10.8)
**Anxiety**	57.4 (12.4)	57.2 (11.3)	57.7 (13.3)
**Perfectionism**	48.2 (8.3)	48.6 (8.9)	47.8 (7.8)
**Social Problems**	54.1 (11.0)	53.7 (10.8)	54.3 (11.2)
**Global scales (T-scores)** †			
**ADHD Index**	56.0 (11.0)	56.1 (11.5)	56.0 (10.5)
**Global Restless-Impulsive**	55.9 (11.3)	56.1 (11.8)	55.8 (10.7)
**Global Emotional Lability**	53.7 (12.7)	53.0 (11.5)	54.3 (13.7)
**Global Index Total**	55.8 (11.8)	55.6 (11.9)	56.0 (11.7)
**DSM-IV Inattentive**	58.1 (10.2)	57.8 (10.5)	58.4 (9.8)
**DSM-IV Hyperactive-Impulsive**	52.5 (10.9)	52.8 (11.4)	52.2 (10.5)
**DSM-IV Total**	56.2 (10.1)	56.1 (10.6)	56.3 (9.7)

Parent rated‡‡ Sub-scales (T-scores)†	Whole sample (n = 295)	Active (n = 148)	Placebo (n = 147)
**Oppositional**	56.9 (12.0)	58.0 (13.2)	55.8 (10.6)
**Cognitive Problems**	59.2 (11.1)	60.3 (11.4)	58.1 (10.7)
**Hyperactivity**	54.3 (9.8)	54.6 (9.8)	54.0 (9.9)
**Anxiety**	52.2 (10.4)	52.2 (11.0)	52.1 (9.9)
**Perfectionism**	48.9 (9.2)	48.4 (9.2)	49.3 (9.2)
**Social Problems**	56.2 (12.2)	56.7 (12.7)	55.7 (11.8)
**Psychosomatic**	55.6 (12.3)	54.7 (11.8)	56.5 (12.7)
**Global scales (T-scores)** †			
**ADHD Index**	57.7 (11.3)	58.6 (11.8)	56.8 (10.8)
**Global Restless-Impulsive**	56.8 (10.8)	57.7 (11.1)	56.0 (10.5)
**Global Emotional Lability**	54.4 (11.8)	55.3 (12.4)	53.4 (11.0)
**Global Index Total**	56.7 (11.1)	57.6 (11.5)	55.8 (10.7)
**DSM-IV Inattentive**	56.1 (10.8)	57.1 (11.2)	55.1 (10.4)
**DSM-IV Hyperactive-Impulsive**	57.2 (11.5)	57.6 (11.5)	56.8 (11.6)
**DSM-IV Total**	57.1 (10.9)	57.9 (11.3)	56.3 (10.5)

* Obtained from the British Ability Scales II.^13^

‡ Obtained from Conners' Teacher Rating Scale (CTRS-L).^15^

‡‡ Obtained from Conners' Parent Rating Scale (CPRS-P).^15^

§ Standard Scores have a mean of 100, sd = 15.

† Standard Scores have a mean of 50 sd = 10. For values above the mean, higher scores indicate more severe difficulties with behaviour and/or attention.

### Did blinding work?

Parent and teacher estimates of group allocation at post-intervention were used to assess the maintenance of blinding. Group comparisons carried out on these estimates showed there were no significant differences between groups, as shown in Table 3.

**Table 3:** Maintenance of Blinding for Parents and Teachers, n (%).

	Actual Treatment Allocation			
	Active		Placebo	
“Guessed” treatment allocation	Parent (n = 135)	Teacher (n = 140)	Parent (n = 145)	Teacher (n = 136)
**Placebo**	87 (64.4%)	85 (60.7%)	87 (60.0%)	84 (61.8%)
**Active**	45 (33.3%)	51 (36.5%)	55 (37.9%)	49 (36.0%)
**Don't know**	3 (2.2%)	4 (2.9%)	3 (2.1%)	3 (2.3%)

All values non-significant for Active versus Placebo using χ2 test.

### Numbers analyzed

Intention-to-treat analyses were carried out on all children randomized (n = 362) and on the pre-planned sub-groups defined by baseline reading ≤20th and 10th centiles (n = 224 and n = 105 respectively) in line with the original protocol. Behavior ratings were the only measures with >15% of the data missing (change scores n = 273 for Teachers, and n = 246 for Parents), so additional per protocol analyses were conducted on these measures.

### Outcomes

#### a) Reading

**Figure 2:**
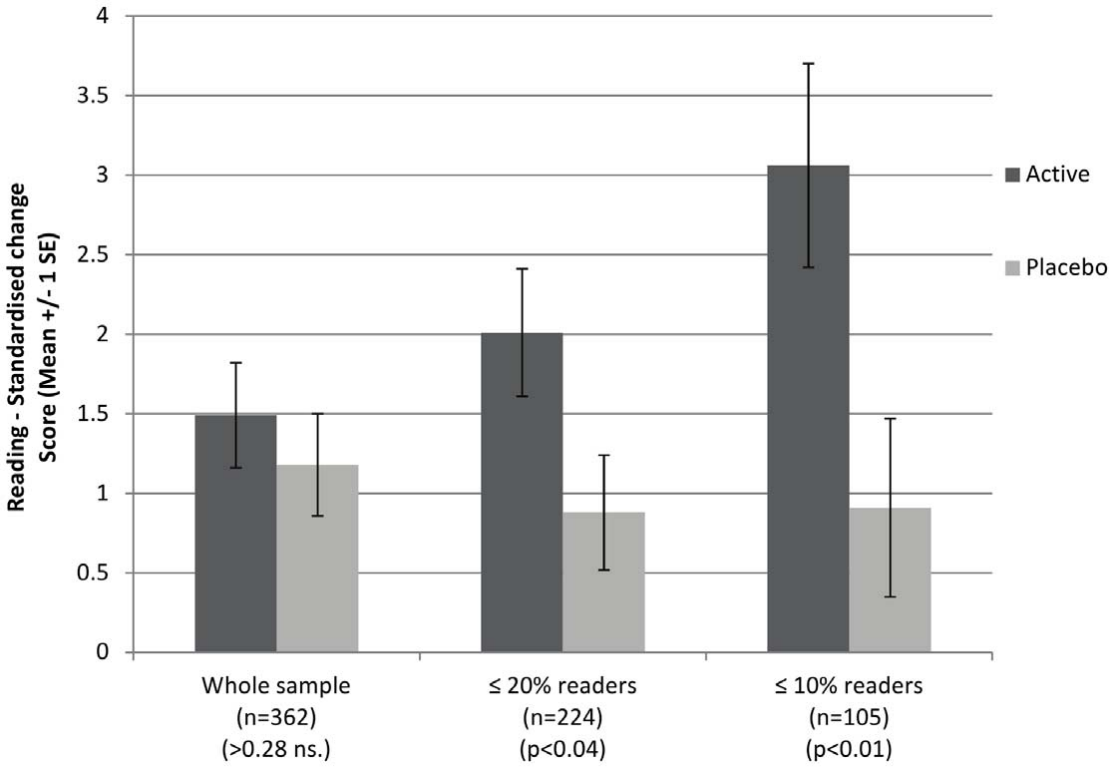
Change in reading scores between baseline and post-intervention.

Standardized reading score data are shown in Table 4, and changes on this measure, which were the primary outcome, are illustrated in Figure 2. The same data expressed as ‘reading ages’ are shown in Table 5.

**Table 4:** Standardized* Reading Scores, means (sd).

	Baseline			Post-Intervention			Change Scores		
	Active	Placebo	P	Active	Placebo	P	Active	Placebo	P
**All randomized (n = 362)**	84.6 (6.6)	84.8 (6.1)	0.937	86.1 (7.0)	86.0 (7.5)	0.895	1.5 (4.4)	1.2 (4.3)	0.279
**Reading ≤20th Centile (n = 224)**	80.6 (5.3)	81.2 (4.8)	0.582	82.6 (6.0)	82.1 (6.1)	0.708	2.0 (4.2)	0.9 (3.9)	0.041
**Reading ≤10th Centile (n = 105)**	75.6 (4.0)	77.5 (3.9)	0.006	78.7 (5.5)	78.4 (5.8)	0.862	3.1 (4.4)	0.9 (4.2)	0.011

* Standardized scores have a mean of 100, sd = 15.

Reading standard scores are derived from the British Ability Scales II.^13^

**Table 5:** Reading Age* and Chronological age (in months), means (sd).

	Baseline			Post-Intervention			Change scores		
	Active	Placebo	P	Active	Placebo	P	Active	Placebo	P
**All randomized (n = 362)**	86.1 (10.0)	87.1 (9.7)	0.331	90.8 (10.1)	91.9 (10.9)	0.307	4.7 (4.7)	4.8 (4.6)	0.856
*Chronological age*	*103.7 (10.0)*	*104.8 (10.1)*							
**Reading ≤20th Centile (n = 224)**	82.5 (9.1)	84.1 (8.6)	0.173	87.4 (9.0)	88.3 (9.4)	0.288	4.9 (4.7)	4.1 (4.0)	0.239
*Chronological age*	*104.6 (9.5)*	*106.1 (9.8)*							
**Reading ≤10th Centile (n = 105)**	77.3 (7.3)	80.8 (7.9)	0.019	83.0 (7.0)	84.7 (8.8)	0.126	5.7 (4.8)	3.8 (4.1)	0.032
*Chronological age*	*104.8 (9.0)*	*107.1 (9.8)*							

* Reading ages are derived from the British Ability Scales II.^13^

Over the 16-week treatment period, very slight improvements in reading were found over and above the gains that would be expected during this time period. For all children randomized (n = 362), the changes in standardized reading scores did not differ by treatment group (Active mean = 1.5, sd = 4.4; Placebo mean = 1.2, sd = 4.3). However, the planned analyses for pre-defined sub-groups of poorer readers did show significant effects of treatment on reading change. For children with baseline reading ≤20^th^ centile (n = 224), improvements were greater for active treatment (Active mean = 2.0, sd = 4.2; Placebo mean = 0.9, sd = 3.9, p<0.04); and for those with baseline reading ≤10^th^ centile (n = 105), the treatment effect was slightly greater (Active mean = 3.1, sd = 4.4; Placebo mean = 0.9, sd = 4.2, p<0.01).

The subgroup analysis was undertaken with an interaction effect of DHA supplementation on reading with the primary sub-group, i.e. those with initial reading ≤20th centile (n = 224). The interaction effect was significant and positive (Interaction: treatment*sub-group 2.152, p< = 0.05; OLS regression with main effects included (treatment and subgroup)). Therefore the treatment effect in the ≤20th centile group can be said to be robust [22].

#### b) Working memory

Data on these measures are provided in Table 6. Post-intervention, standardized scores on Recall of Digits Forward improved slightly in the sample as a whole (mean change score = 1.4, sd = 6.0) whereas there was no change in Recall of Digits Backward (mean change score = 0.0, sd = 7.3). In each case there were no significant group differences on the primary outcome of change scores, although post-intervention scores on Recall of Digits Forward were higher in the active treatment group as a whole (Active mean = 42.6, sd = 8.4, Placebo mean = 41.2, sd = 7.7, p<0.04).

**Table 6:** Standardized Working Memory Scores (Recall of Digits Forward and Backward)†, means (sd).

	Baseline T Score (sd)			Post-Intervention T Score (sd)			Change Scores, T Score (sd)		
	Active	Placebo	P	Active	Placebo	P	Active	Placebo	P
**RECALL OF DIGITS FORWARD**									
**All randomized (n = 362)**	41.0 (8.2)	40.0 (7.7)	0.174	42.6 (8.4)	41.2 (7.7)	0.037	1.6 (6.2)	1.2 (5.7)	0.419
**Reading ≤20th Centile (n = 224)**	39.8 (8.0)	39.9 (8.6)	0.812	41.9 (8.5)	40.5 (8.0)	0.104	2.0(6.8)	0.7 (5.6)	0.069
**Reading ≤10th Centile (n = 105)**	39.4 (7.3)	39.1 (9.9)	0.607	41.9 (8.0)	40.2 (9.3)	0.132	2.5 (7.2)	1.1 (6.0)	0.247
**RECALL OF DIGITS BACKWARD**									
**All randomized(n = 362)**	44.0 (6.4)	44.0 (7.1)	0.767	44.3 (7.1)	43.7 (7.0)	0.621	0.4 (7.3)	−0.3 (7.4)	0.204
**Reading ≤20th Centile (n = 224)**	43.7 (6.1)	43.5 (7.5)	0.841	44.3 (7.0)	42.8 (7.7)	0.396	0.6 (6.8)	−0.7 (7.3)	0.173
**Reading ≤10th Centile (n = 105)**	42.9 (5.0)	43.2 (7.2)	0.837	44.0 (7.5)	42.3 (8.6)	0.473	1.0 (6.5)	−0.9 (8.2)	0.187

† T scores have a mean of 50, sd = 10 and are derived from the British Ability Scales II.^13^.

**Figure 3:**
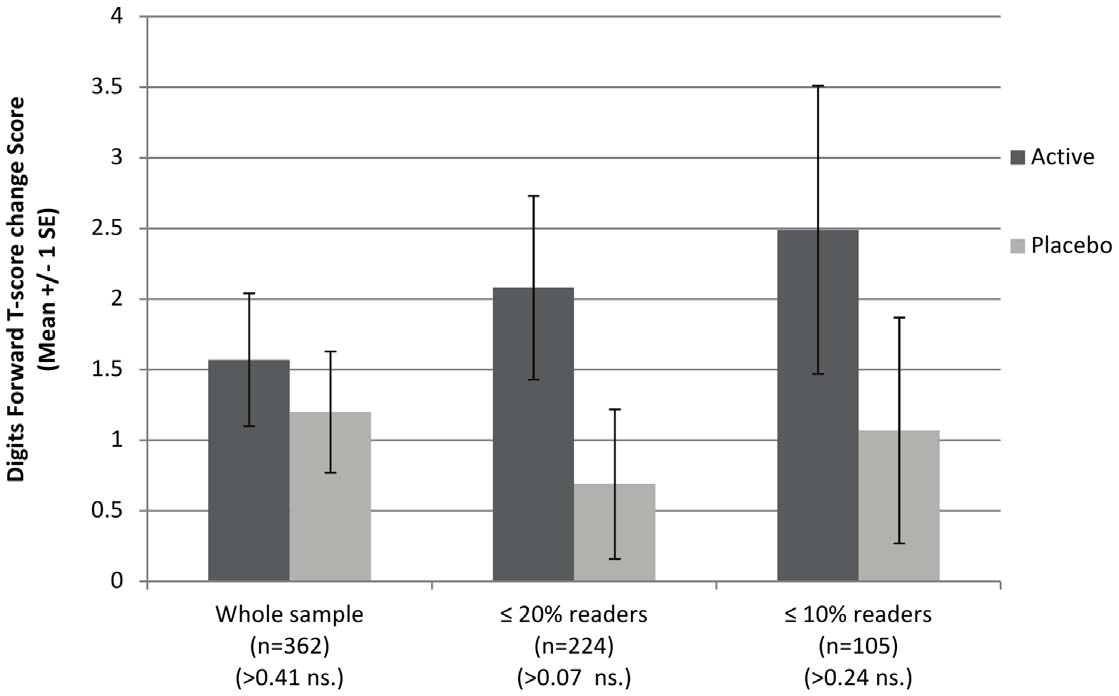
Change in working memory scores (recall of digits forward) between baseline and post-intervention.

**Figure 4:**
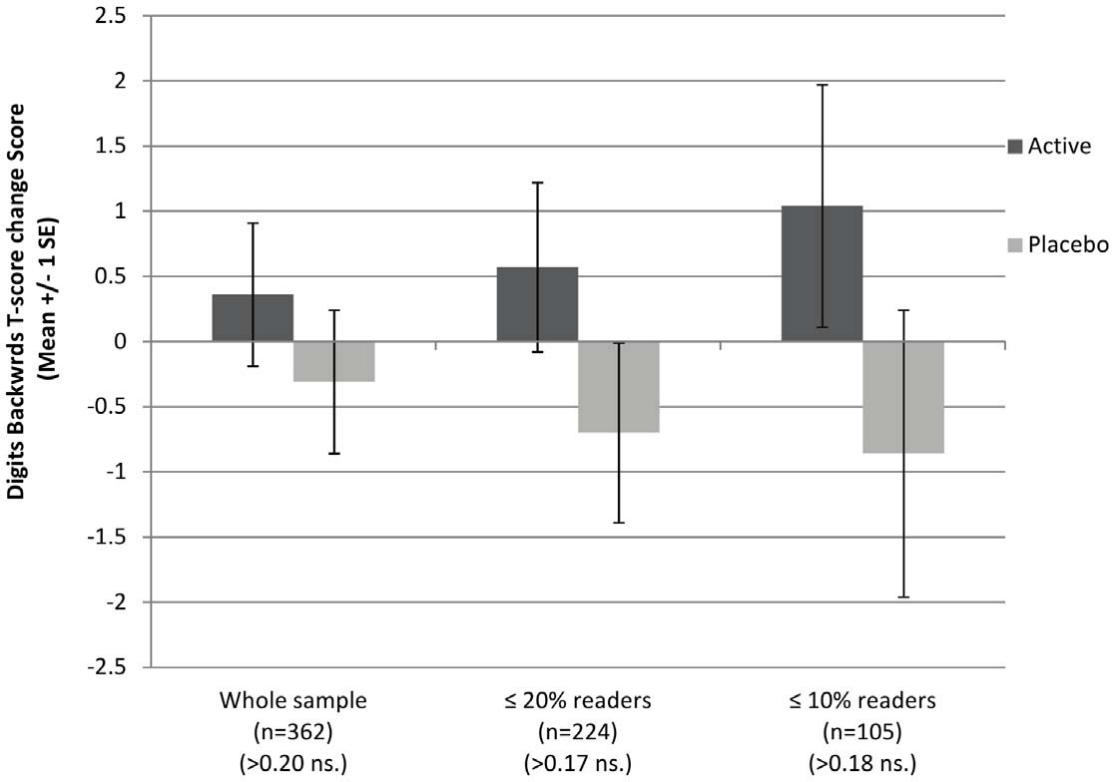
Change in working memory scores (recall of digit backwards) between baseline and post-intervention.

There were no significant effects of treatment on the working memory change scores in the pre-planned sub-groups with baseline reading ≤20^th^ or 10^th^ centiles. There was, however, a suggestion that the slight group difference in favor of active treatment increased with the degree of reading impairment, as illustrated in Figures 3 and 4.

#### c) Behavior – Parent and Teacher ratings

For the sample as a whole, behavior ratings from parents were lower post-intervention than at baseline for almost all scales, as shown in Table 7 (ITT) and Table 8 (per protocol) Teacher ratings, however, showed minimal changes over the 16-week treatment period, as shown in Table 9 (ITT) and Table 10 (per protocol).

**Table 7:** Standardized* Behavior Scores - Parent rated† (intention to treat), means (sd).

	Baseline		Post-Intervention		Change Score		
	Active (n = 180)	Placebo (n = 182)	Active (n = 180)	Placebo (n = 182)	Active (n = 180)	Placebo (n = 182)	P
**Oppositional**	57.7 (12.0)	55.4 (9.5)	54.4 (10.8)	55.0 (9.6)	−3.2 (10.1)	−0.4 (8.1)	0.004
**Cognitive Problems**	59.7 (10.4)	57.7 (9.6)	56.0 (9.2)	55.7 (9.4)	−3.7 (8.5)	−2.0 (7.6)	0.055
**Hyperactivity**	54.1 (8.9)	53.5 (9.0)	51.2 (8.6)	52.2 (7.8)	−2.9 (7.2)	−1.2 (7.1)	0.007
**Anxiety**	51.7 (10.0)	51.7 (8.9)	47.9 (7.6)	49.9 (9.2)	−3.8 (8.5)	−1.8 (8.9)	0.074
**Perfectionism**	48.0 (8.3)	48.6 (8.4)	46.1 (6.9)	47.3 (7.7)	−1.9 (6.9)	−1.4 (7.4)	0.333
**Social Problems**	55.9 (11.6)	54.8 (10.7)	52.9 (9.8)	53.2 (10.4)	−3.0 (10.0)	−1.6 (10.8)	0.164
**Psychosomatic**	54.4 (10.7)	55.9 (11.5)	50.4 (9.4)	53.0 (10.7)	−4.0 (10.2)	−2.8 (11.2)	0.168
**ADHD Index**	58.2 (10.7)	56.7 (9.8)	53.7 (9.5)	54.1 (9.7)	−4.5 (8.9)	−2.6 (7.4)	0.042
**Global Restless-Impulsive**	57.6 (10.0)	55.8 (9.4)	52.8 (9.8)	53.6 (9.6)	−4.8 (8.6)	−2.3 (8.2)	0.001
**Global Emotional Lability**	54.7 (11.3)	53.2 (9.9)	51.5 (10.8)	53.0 (9.7)	−3.1 (9.5)	−0.2 (9.3)	0.001
**Global Index Total**	57.2 (10.5)	55.4 (9.6)	52.9 (10.2)	53.7 (9.5)	−4.3 (8.8)	−1.8 (8.2)	0.001
**DSM-IV Inattention**	56.7 (10.2)	54.7 (9.3)	53.5 (9.3)	52.9 (9.4)	−3.2 (8.3)	−1.8 (7.5)	0.087
**DSM-IV Hyperactive-Impulsive**	57.2 (10.5)	56.3 (10.5)	53.4 (10.0)	54.0 (9.6)	−3.8 (8.3)	−2.2 (8.2)	0.021
**DSM-IV Total ADHD**	57.4 (10.3)	56.1 (9.4)	53.6 (9.7)	54.0 (9.5)	−3.8 (8.1)	−2.1 (7.4)	0.031

* T scores have a mean of 50, sd = 10. For values above the mean, higher scores indicate more severe difficulties with behaviour and/or attention.

† Behaviour measures are derived from the Conners' Teacher Rating Scale.^15^

**Table 8:** Standardized* Behavior Scores - Parent rated† (per protocol), means (sd).

	Baseline		Post-Intervention		Change Score		
	Active (n = 148)	Placebo (n = 147)	Active (n = 145)	Placebo (n = 148)	Active (n = 121)	Placebo(n = 128)	P
**Oppositional**	58.0 (13.2)	55.8 (10.6)	55.0 (11.9)	55.3 (10.6)	−2.1 (8.9)	−0.3 (6.8)	0.117
**Cognitive Problems**	60.3 (11.4)	58.1 (10.7)	56.2 (10.2)	55.6 (10.5)	−3.46 (7.9)	−2.61 (6.5)	0.246
**Hyperactivity**	54.6 (9.8)	54.0 (9.9)	51.8 (9.5)	52.5 (8.6)	−2.45 (6.7)	−1.32 (5.9)	0.073
**Anxiety**	52.2 (11.0)	52.1 (9.9)	48.3 (8.4)	50.6 (10.1)	−4.1 (8.5)	−2.0 (7.7)	0.052
**Perfectionism**	48.4 (9.2)	49.3 (9.2)	46.7 (7.6)	47.8 (8.5)	−2.0 (6.7)	−1.6 (7.1)	0.459
**Social Problems**	56.7 (12.7)	55.7 (11.8)	53.6 (10.8)	53.9 (11.4)	−2.4 (9.4)	−1.4 (9.7)	0.522
**Psychosomatic**	54.7 (11.8)	56.5 (12.7)	51.0 (10.4)	53.7 (11.8)	−3.9 (9.9)	−3.0 (10.2)	0.362
**ADHD Index**	58.6 (11.8)	56.8 (10.8)	54.1 (10.5)	54.3 (10.8)	−3.9 (7.8)	−2.6 (6.2)	0.073
**Global Restless-Impulsive**	57.7 (11.1)	56.0 (10.5)	53.5 (10.8)	53.9 (10.7)	−4.1 (7.5)	−2.2 (6.8)	0.018
**Global Emotional Lability**	55.3 (12.4)	53.4 (11.0)	52.4 (11.9)	53.2 (10.7)	−2.4 (7.7)	−1.0 (8.3)	0.028
**Global Index Total**	57.6 (11.5)	55.8 (10.7)	53.6 (11.3)	54.1 (10.5)	−3.8 (7.1)	−2.0 (6.8)	0.018
**DSM-IV Inattention**	57.111.2)	55.1 (10.4)	53.810.4)	53.3 (10.3)	−2.75 (7.4)	−2.12 (6.1)	0.104
**DSM-IV Hyperactive-Impulsive**	57.6 (11.5)	56.8 (11.6)	53.9 (11.1)	54.5 (10.6)	−3.1 (7.5)	−2.3 (6.4)	0.425
**DSM-IV Total ADHD**	57.9 (11.3)	56.3 (10.5)	54.2 (10.7)	54.3 (10.5)	−3.1 (7.1)	−2.3 (5.9)	0.219

* T scores have a mean of 50, sd = 10. For values above the mean, higher scores indicate more severe difficulties with behaviour and/or attention.

† Behaviour measures are derived from the Conners' Teacher Rating Scale.^15^

**Table 9:** Standardized* Behavior Scores - Teacher rated† (intention to treat), means (sd).

	Baseline		Post-Intervention		Change Score		
	Active (n = 180)	Placebo (n = 182)	Active (n = 180)	Placebo (n = 182)	Active (n = 180)	Placebo (n = 182)	P
**Oppositional**	53.7 (12.0)	56.0 (13.8)	52.7 (12.1)	53.8 (11.5)	−1.1 (10.6)	−2.2 (10.7)	0.701
**Cognitive Problems**	61.7 (9.2)	61.4 (8.6)	60.0 (8.7)	60.0 (8.0)	−1.7 (7.7)	−1.4 (8.5)	0.765
**Hyperactivity**	53.0 (10.3)	53.2 (10.5)	52.4 (9.5)	51.1 (9.0)	−0.6 (7.3)	−2.1 (9.2)	0.175
**Anxiety**	57.0 (10.6)	57.5 (12.9)	54.7 (11.1)	54.7 (10.9)	−2.3 (10.6)	−2.8 (11.4)	0.584
**Perfectionism**	48.0 (8.5)	47.6 (7.6)	48.2 (7.8)	46.7 (6.5)	0.2 (6.5)	−0.8 (7.4)	0.033
**Social Problems**	53.3 (10.2)	54.1 (11.0)	52.4 (9.5)	52.5 (9.9)	−0.9 (9.0)	−1.6 (9.8)	0.324
**ADHD Index**	55.8 (10.7)	55.9 (10.2)	53.8 (9.9)	54.9 (8.9)	−2.1 (8.2)	−1.0 (9.2)	0.134
**Global Restless-Impulsive**	56.0 (11.1)	55.8 (10.4)	54.3 (10.6)	54.3 (9.2)	−1.7 (8.6)	−1.4 (9.5)	0.714
**Global Emotional-Lability**	52.1 (11.0)	53.8 (13.4)	51.2 (10.2)	51.5 (10.7)	−0.9 (10.8)	−2.3 (11.2)	0.901
**Global Index Total**	55.3 (11.2)	55.9 (11.4)	53.7 (10.7)	54.4 (9.6)	−1.5 (9.1)	−1.5 (9.8)	0.569
**DSM-IV Inattention**	57.7 (9.8)	58.4 (9.5)	56.0 (9.8)	56.2 (8.8)	−1.7 (8.2)	−2.2 (9.1)	0.651
**DSM-IV Hyperactive-Impulsive**	52.3 (10.7)	52.0 (10.2)	50.8 (9.5)	50.2 (8.9)	−1.5 (7.7)	−1.8 (8.7)	0.729
**DSM-IV Total ADHD**	55.8 (9.9)	56.3 (9.4)	54.3 (9.6)	54.3 (8.2)	−1.5 (7.5)	−2.1 (8.6)	0.821

* T scores have a mean of 50, sd = 10. For values above the mean, higher scores indicate more severe difficulties with behaviour and/or attention.

† Behavior measures are derived from the Conners' Teacher Rating Scale.^15^

**Table 10:** Standardized* Behavior Scores - Teacher rated† (per protocol), means (sd).

	Baseline		Post-Intervention		Change Score		
	Active (n = 158)	Placebo (n = 171)	Active (n = 150)	Placebo (n = 147)	Active (n = 134)	Placebo (n = 139)	P
**Oppositional**	54.5 (12.6)	56.3 (14.2)	54.0 (12.9)	55.2 (12.5)	−0.1 (8.1)	−0.8 (9.0)	0.578
**Cognitive Problems**	61.8 (9.8)	61.5 (8.9)	60.1 (9.5)	60.2 (8.9)	−1.4 (7.3)	−1.0 (8.0)	0.702
**Hyperactivity**	53.4 (11.0)	53.4 (10.8)	52.9 (10.3)	51.8 (9.9)	0.2 (6.6)	−0.9 (7.3)	0.257
**Anxiety**	57.2 (11.3)	57.7 (13.3)	55.1 (12.1)	55.1 (12.1)	−2.3 (9.3)	−1.1 (10.4)	0.121
**Perfectionism**	48.6 (8.9)	47.8 (7.8)	48.9 (8.4)	47.4 (7.1)	0.6 (5.8)	−0.4 (6.7)	0.107
**Social Problems**	53.7 (10.8)	54.3 (11.2)	53.1 (10.3)	53.5 (10.8)	−0.8 (7.9)	−0.6 (9.5)	0.755
**ADHD Index**	56.1 (11.5)	56.0 (10.5)	54.3 (10.8)	55.1 (9.9)	−1.4 (7.5)	−0.1 (8.3)	0.104
**Global Restless-Impulsive**	56.1 (11.8)	55.8 (10.7)	54.9 (11.5)	54.9 (10.2)	−0.7 (7.9)	−0.1 (8.5)	0.597
**Global Emotional Lability**	53.0 (11.5)	54.3 (13.7)	52.3 (10.9)	52.8 (11.5)	0.1 (8.8)	−0.5 (9.0)	0.949
**Global Index Total**	55.6 (11.9)	56.0 (11.7)	54.5 (11.6)	54.7 (10.6)	−0.5 (8.0)	−0.3 (8.3)	0.653
**DSM-IV Inattention**	57.8 (10.5)	58.4 (9.8)	56.5 (10.6)	56.5 (9.7)	−1.2 (7.9)	−1.4 (8.5)	0.817
**DSM-IV Hyperactive-Impulsive**	52.8 (11.4)	52.2 (10.5)	51.5 (10.3)	50.9 (9.7)	−0.6 (6.8)	−0.6 (6.9)	0.986
**DSM-IV Total ADHD**	56.1 (10.6)	56.3 (9.7)	54.8 (10.5)	54.5 9.1)	−0.9 (7.0)	−1.2 (7.5)	0.951

* T scores have a mean of 50, sd = 10. They are symptom scales so for values above the mean, higher scores indicate more severe difficulties with behaviour and/or attention.

† Behavior measures are derived from the Conners' Teacher Rating Scale.^15^

### Parent-rated behavior

The ITT analyses showed that behavioral improvements as rated by parents were greater for Active treatment over Placebo on six of the seven CPRS-L Global scales (Table 7). Their means (sds) were as follows: ADHD Index (Active −4.5 (8.9), Placebo −2.6 (7.4), p<0.04), Restless-Impulsive (Active −4.8 (8.6), Placebo −2.3 (8.2), p<0.01), Emotional Lability (Active −3.1 (9.5), Placebo −0.2 (9.3), p<0.01), Global Index Total (Active −4.3 (8.8), Placebo −1.8 (8.2), p<0.01), DSM-IV Hyperactive-Impulsive (Active −3.8 (8.3), Placebo −2.2 (8.2), p<0.02), and DSM IV Total (Active −3.8 (8.1), Placebo −2.1 (7.4), p<0.03). Significant differences in favor of Active treatment were also found for two of the seven CPRS-L sub-scales: Oppositional (Active −3.2 (10.1), Placebo −0.4 (8.1), p<0.01), and Hyperactivity (Active −2.9 (7.2), Placebo −1.2 (7.1), p<0.01).

In the per protocol analyses (n = 247), a similar pattern of results was found (Table 8), although differences reached statistical significance only for Anxiety (Active −4.1 (8.5), Placebo −2.0 (7.7, p = <0.05), Global Restless-Impulsive (Active −4.1 (7.5), Placebo −2.2 (6.8), p<0.02), Global Emotional Lability (Active −2.4 (7.7), Placebo −1.0 (8.3), p<0.03) and Global Index Total (Active −3.8 (7.1), Placebo −2.0 (6.8) p<0.02).

No significant effects of treatment were found within the sub-groups defined by severity of reading impairment at baseline. (These data are not shown, but can be provided on request).

### Teacher-rated behavior

The ITT analyses showed no significant differences in behavior change scores between Active and Placebo groups on any of the CTRS-L sub-scales or global scales, with the exception of the ‘Perfectionism’ sub-scale, on which both groups scored below the normal population mean at baseline and post-intervention. (Active mean = 0.2, sd = 6.5, Placebo mean = −0.8, sd = 7.4, p<0.03). In the per protocol analyses (n = 273), no group differences were significant.

No significant effects of treatment on teacher-rated behavior change were found in the sub-groups defined by severity of reading impairment at baseline. (These data are not shown, but can be provided on request).

### Adverse events

The DHA supplement used is generally regarded as safe (GRAS) and so no stopping guidelines were put in place except in the case of serious adverse events. As expected, there were none in the course of this trial. The parents of three children reported minor adverse events during the intervention period and so they discontinued treatment, but no unblinding was required. The adverse events were: asthma symptoms (Active), nettle rash (Placebo) and disruptive behavior (Active). The parent of one child in the Active group reported hair loss 6 weeks after completing the study.

#### Reported side effects

No group differences were found for 16 of the 17 potential side effects assessed by the Barkley scale. However, children in the Placebo group (n = 146) were reported to have more insomnia or trouble sleeping than those taking the Active supplement (n = 140). Means (sds) for this item were 1.4 (2.2) and 1.0 (2.0) respectively (p = <0.03).

### Compliance

Counts of capsules returned by schools indicated mean compliance of approximately 75% and this did not differ between Active (n = 138) and Placebo groups (n = 157). (From 200 capsules allocated to schools for each child, quantities returned were: Active mean = 50.0, sd = 51.8) and Placebo Mean = 42.0, sd = 39.5). Of the 50 capsules allocated to parents for non-school days, more than 50% of data were missing and so these are not reported.

## Discussion

In this randomized controlled trial we investigated the effects of dietary supplementation for 16 weeks with 600 mg/day of the omega-3 fatty acid DHA or placebo in 362 healthy school children mainly aged 7–9 years who were initially underperforming in reading (≤33^rd^ centile). Our primary outcomes were reading, working memory, and behavior (ADHD-type symptoms) as rated by both parents and teachers, all of which are known to be important for children's future educational and occupational achievement.

### Outcomes

#### (a) Reading

The effects of DHA on children's reading progress were found to vary with their initial reading performance. No treatment effect was found in the sample as a whole (selected for initial reading ≤33^rd^ centile). However, small but significant benefits from DHA supplementation were seen in the 224 children whose initial reading performance was ≤20^th^ centile, (the target population in our original study design), and these benefits were more pronounced in those children whose initial reading performance was ≤10^th^ centile.

The practical implications of these findings are best illustrated by the reading age scores (derived from the same data as the age-standardized reading scores, and shown in Table 5). In children with initial reading performance ≤20^th^ centile, active treatment was associated with an *additional* 0.8 months mean increase in reading age change scores compared with placebo, while in those initially reading ≤10^th^ centile, the *additional* reading age gain from treatment was 1.9 months. As reading ages would typically be expected to increase by 4 months over the 16-week treatment period, this means that the gains from DHA supplementation in these sub-groups of poorer readers were around 20% and 50% greater, respectively, than would normally be expected, helping these children to catch up with their peer group.

Only two previous studies of omega-3 supplementation in UK children have involved reading as an outcome measure. In an unselected sample of typically developing children aged 8–10 years who were reading normally for their age, no treatment effects were found [23]. By contrast, highly significant benefits were found in children with Developmental Coordination Disorder, whose reading performance was initially well below the level expected for their age [7]. Our results are broadly compatible with both these findings, in that we only found benefits for reading in those children whose initial performance was at or below the 20^th^ centile. Recently, a small study from Australia found no overall treatment effect of omega-3 on reading, although this trial was small and underpowered, and involved children selected for ADHD symptoms [24]. Significant correlations were found, however, between increases in blood DHA and improvements in reading; and these associations were more pronounced in the subgroup who also had reading difficulties.

#### (b) Working Memory

Group differences did not reach significance for the primary outcome of change scores on the working memory measures (Recall of Digits Forward and Backward), although the mean scores post-intervention for children receiving DHA were significantly higher on Recall of Digits Forward (tapping auditory sequential verbal memory).

The children in this study were not selected for working memory problems, but their initial scores on these measures were found to be 0.5–1 sd below population norms (consistent with the importance of working memory in the development of reading skills). Thus there was some room for improvement on these measures in our sample. This was not the case in the only other RCT of mainstream schoolchildren to investigate omega-3 for working memory, which found no effects of supplementation using similar measures [23]. In the present study, the subgroup analyses suggested that any improvements in working memory might be greater in children with poorer initial reading performance, but further studies would be need to explore this possibility.

#### (c) Behavior

Parent ratings of the children's behavior showed significant reductions on most symptom scales at the 16-week follow-up, i.e. there was a significant placebo effect. Despite this, the ITT analyses showed significant effects of DHA over placebo for the sample as a whole on 8 of the 14 scales, assessing a range of ADHD-type symptoms. These included sub-scales assessing hyperactivity and oppositional behaviour, and global scales tapping emotional lability (mood swings) and restless-impulsive behaviour as well as total ADHD-type symptoms. These findings are in keeping with those from previous trials of omega-3 LC-PUFA supplementation in children with various developmental disorders of behavior and learning [25], [26] it is notable that similar effects were found here in normal healthy children, even though their behavioral problems pre-treatment were within the normal population range.

By contrast, teachers' ratings of the children's behavior showed minimal changes during the 16-week treatment period, and no differences between treatment groups. Disparities between teacher and parent ratings of child behavior are extremely common (which is why a formal clinical diagnosis of ADHD requires consistency across different settings). It is perhaps unsurprising that parents might be more sensitive than teachers or other professionals to any changes in their child's behavior over a short intervention period, as this has been found in controlled studies of other nutritional interventions for child behaviour [27] as well as in other trials of omega-3 supplementation [26]. However, teacher ratings have proved sensitive to behavioral change in some of the previous studies of omega-3 for ADHD-type symptoms in children [7], [23].

It is possible that missing data from parent ratings in our current study could have introduced an element of positive bias, but we think this unlikely. The findings are in line with the accumulating evidence that supplementation with omega-3 is of modest benefit in reducing ADHD-type symptoms in children [6], [28]. They also broaden this evidence from clinical groups with varying types of developmental disorders, and other children whose behavioral problems are above average, to healthy children whose behavioral problems fall within the normal population range.

### Generalizability

The context in which this trial was carried out was mainstream UK schools, where the influence of the researchers was low and contact with parents was indirect and minimal, whereas most previous trials have involved children with specific behavioral and/or learning difficulties recruited either from clinics or from direct advertisement to parents. This classifies it as an “effectiveness” - i.e. ‘real-world’ study - rather than an “efficacy” study, and provides reasonable grounds to trust the generalizability of these data to other mainstream school populations, albeit with some caveats.

Generalizability to *all* children aged 7–9 years attending mainstream UK schools is clearly limited, as our sample was pre-selected for underperformance in reading. In terms of socioeconomic status, the children in this study were fairly representative of the general population of England, as the percentage receiving free school meals was comparable to national figures (20.2% vs 18.6%). However, ethnic minorities were under-represented (91.2% were white, compared with 77.7% nationally), primarily because our study required that children use English as their first language at home. The percentage of boys was also slightly elevated (53% vs 51% nationally), reflecting the fact that in this age range, boys are typically more likely than girls to be underperforming in reading.

### Implications for Research and Practice

Various implementation issues concerning dose and formulation, delivery and uptake may have a bearing on these results and therefore merit brief consideration.

The dose we selected (600 mg/day) was comparable to those used in most other trials of long-chain omega-3 for child behavior and/or learning to date [6], and only slightly higher than the intake of 500 mg/day recommended for general cardiovascular health in adults by leading scientists in the field [29]. Higher doses might possibly yield more substantial treatment effects, but this would need to be investigated via systematic dose-ranging studies, because intakes of individual nutrients may not relate in any simple linear fashion to health or performance outcomes [30].

The formulation we used provided only DHA, whereas most previous studies have used varying combinations of DHA, EPA and sometimes other fatty acids. A recent meta-analysis found that treatment effects on ADHD-type symptoms increased with the proportion of EPA (and thus decreased with the proportion of DHA) in the supplements used [6]. This analysis was based on only 10 small and highly heterogeneous trials, however, and direct comparisons would be needed to evaluate the relative merits of different formulations with any degree of certainty.

The efficiency of delivery and uptake can also influence results in any intervention study. The supplements were provided to children on school days by a range of different staff at the 74 schools involved. Counts of capsules returned from schools suggested that satisfactory compliance with the treatment was achieved in these settings, but these data were not complete. Similarly, the parents who gave the children their supplies during weekends and other non-school days will have varied in their motivation and adherence to the treatment schedule; compliance data here were unfortunately impossible to obtain, in line with other studies.

This study provides the first evidence that dietary supplementation with the omega-3 DHA might improve both the behavior and the learning of healthy children from the general school population. The supplement was found to be safe and well tolerated, as expected. The benefits we found for reading were only evident amongst those children whose initial reading performance fell within the lowest 20% of the normal distribution, suggesting that for this particular outcome measure, DHA supplementation should be regarded as a targeted intervention for the poorest readers rather than a universal one. For behavior, however, it is possible that the benefits may have broader applicability, as reductions in parents' ratings of ADHD-type symptoms were evident across the entire sample studied here, despite the relatively mild nature of these children's initial behavior problems. The current results thus extend the previous findings in this area from clinically-defined groups to healthy but underperforming children from the general school population, and suggest that dietary deficiencies of DHA might have subtle behavioral effects on children in general, as has been shown for some combinations of artificial food additives [31].

Dietary intakes of long-chain omega-3 in modern western-type diets are widely acknowledged to be sub-optimal, both for general physical health and for mental health and performance [32]. Similarly, the importance of early intervention for behavior and literacy problems in children is widely acknowledged to be more effective and less costly than interventions later in life, by which time such problems can become compounded and the life chances of these children further compromised [11]. These findings clearly require replication, but we suggest that future studies should adopt a similar focus on the most vulnerable groups within the general population, ideally using additional measures to assess the relative costs and benefits of this kind of dietary supplementation.

## Supporting Information

Protocol S1 **Study protocol.**
(DOC)

Checklist S1 **CONSORT checklist.**
(DOC)

## References

[1] Simopoulos A (2002) The importance of the ratio of omega-6/omega-3 essential fatty acids. Biomed Pharmacother 56: 365–379. 12442909 10.1016/s0753-3322(02)00253-6

[2] Brenna JT, Salem N, Sinclair AJ, Cunnane SC (2009) Alpha-Linolenic acid supplementation and conversion to n-3 long-chain polyunsaturated fatty acids in humans. Prostaglandins Leukot Essent Fatty Acids 80: 85–91. 19269799 10.1016/j.plefa.2009.01.004

[3] Schuchardt JP, Huss M, Stauss-Grabo M, Hahn A (2010) Significance of long-chain polyunsaturated fatty acids (PUFAs) for the development and behaviour of children. Eur J Pediatr 169: 149–164. 19672626 10.1007/s00431-009-1035-8

[4] Ryan AS, Astwood JD, Gautier S, Kuratko CN, Nelson EB, et al (2010) Effects of long-chain polyunsaturated fatty acid supplementation on neurodevelopment in childhood: a review of human studies. Prostaglandins Leukot Essent Fatty Acids 82: 305–314. 20188533 10.1016/j.plefa.2010.02.007

[5] Richardson AJ (2006) Omega-3 fatty acids in ADHD and related neurodevelopmental disorders. Int Rev Psychiatry 18: 155–172. 16777670 10.1080/09540260600583031

[6] Bloch MH, Qawasmi A (2011) Omega-3 fatty acid supplementation for the treatment of children with attention-deficit/hyperactivity disorder symptomatology: systematic review and meta-analysis. J Am Acad Child Adolesc Psychiatry 50: 991–1000. 21961774 10.1016/j.jaac.2011.06.008 PMC3625948

[7] Richardson AJ, Montgomery P (2005) The Oxford-Durham study: a randomized, controlled trial of dietary supplementation with fatty acids in children with developmental coordination disorder. Pediatrics 115: 1360–1366. 15867048 10.1542/peds.2004-2164

[8] Ells L, Hillier F, Summerbell C (2006) A systematic review of the effect of nutrition, diet and dietary change on learning, education and performance of children of relevance to UK schools. Middlesborough: University of Teeside.

[9] Department of Education website. Available: www.education.gov.uk/rsgateway/DB/STR/d001032/osr20-2011.pdf. Accessed 2012 Jul 17.

[10] Her Majesty's Stationery Office website. Available: www.hm-treasury.gov.uk/d/leitch_finalreport051206.pdf. Accessed 2012 Jul 17.

[11] Heckman J, Masterov D (2007) The Productivity Argument for Investing in Young Children. Appl Econ Perspect Pol 29: 446–493.

[12] Gathercole SE, Alloway TP, Willis C, Adams A-M (2006) Working memory in children with reading disabilities. J Exp Child Psychol 93: 265–281. 16293261 10.1016/j.jecp.2005.08.003

[13] Nevo E, Breznitz Z (2011) Assessment of working memory components at 6 years of age as predictors of reading achievements a year later. J Exp Child Psychol 109: 73–90. 21115182 10.1016/j.jecp.2010.09.010

[14] Collishaw S, Gardner F, Maughan B, Scott J, Pickles A (2012) Do historical changes in parent-child relationships explain increases in youth conduct problems? J Abnorm Child Psychol 40: 119–132. 21789521 10.1007/s10802-011-9543-1

[15] Elliott B, Smith P, McCulloch K (1997) British Ability Scales Second Edition (BAS II): technical manual. Windsor: NFER-Nelson.

[16] Department of Education website. Available: https://www.education.gov.uk/publications/standard/publicationDetail/Page1/QCA/99/457. Accessed 2012 Jul 17.

[17] Conners CK (1989) Conners' Rating Scales Manual. North Tonawanda, NY: Multi-Health Systems.

[18] Hobbs G, Vignoles A (2010) Is children's free school meal ‘eligibility’ a good proxy for family income? Brit Educ J 36: 673–690.

[19] Barkley RA, McMurray MB, Edelbrook CS, Robbins K (1990) Side effects of methylphenidate in children with attention deficit hyperactivity disorder: a systemic, placebo-controlled trial. Pediatrics 86: 184–174. 2196520

[20] Crowe FL, Skeaff CM, Green TJ, Gray AR (2008) Serum n-3 long-chain PUFA differ by sex and age in a populaiton-based survey of New Zealand adolescents and adults. Br J Nutr 99: 168–174. 17678566 10.1017/S000711450779387X

[21] Sun X, Briel M, Busse JW, You JJ, Akl EA, et al (2012) Credibility of claims of subgroup effects in randomised controlled trials: systematic review. BMJ 344 doi 10.1136/bmj.e1553. 10.1136/bmj.e1553 22422832

[22] Brookes ST, Whitely E, Egger M, Smith GD, Mulheran PA, et al (2004) Subgroup analyses in randomized trials: risks of subgroup-specific analyses; power and sample size for the interaction test. J Clin Epidemiol 57: 229–236. 15066682 10.1016/j.jclinepi.2003.08.009

[23] Kirby A, Woodward A, Jackson S, Wang Y, Crawford MA (2010) A double-blind placebo-controlled study investigating the effects of omega-3 supplementation in children aged 8–10 years from a mainstream school population. Res Dev Disabil 31: 718–730. 20171055 10.1016/j.ridd.2010.01.014

[24] Milte CM, Parletta N, Buckley JD, Coates AM, Young RM, et al (2012) Eicosapentaenoic and docosahexaenoic acids, cognition, and behavior in children with attention-deficit/hyperactivity disorder: A randomized controlled trial. Nutrition 28: 670–677. 22541055 10.1016/j.nut.2011.12.009

[25] Richardson AJ, Puri BK (2002) A randomized double-blind, placebo-controlled study of the effects of supplementation with highly unsaturated fatty acids on ADHD-related symptoms in children with specific learning difficulties. Prog Neuropsychopharmacol Biol Psychiatry 26: 233–239. 11817499 10.1016/s0278-5846(01)00254-8

[26] Sinn N, Bryan J (2007) Effect of supplementation with polyunsaturated fatty acids and micronutrients on learning and behavior problems associated with child ADHD. J Dev Behav Pediatr 28: 82–91. 17435458 10.1097/01.DBP.0000267558.88457.a5

[27] Schab DW, Trinh NH (2004) Do artificial food colors promote hyperactivity in children with hyperactive syndromes? A meta-analysis of double-blind placebo-controlled trials. J Dev Behav Pediatr 25: 423–434. 15613992 10.1097/00004703-200412000-00007

[28] Richardson AJ (2012) Review: omega-3 fatty acids produce a small improvement in ADHD symptoms in children compared with placebo. Evid Based Ment Health 15: 46. 22345102 10.1136/ebmental-2011-100523

[29] International Society for the Study of Fatty Acids and Lipids website. Available: www.issfal.org/statements/pufa-recommendations/statement-3. Accessed 2012 Jul 17.

[30] Verkerk AO, den Ruijter HM, de Jonge N, Coronel R (2009) Fish oil curtails the human action potential dome in a heterogeneous manner: implication for arrhythmogenesis. Int J Cardiol 132: 138–140. 18037517 10.1016/j.ijcard.2007.07.145

[31] McCann D, Barrett A, Cooper A, Crumpler D, Dalen L, et al (2007) Food additives and hyperactive behaviour in 3-year-old and 8/9-year-old children in the community: a randomised, double-blinded, placebo-controlled trial. Lancet 370: 1560–1567. 17825405 10.1016/S0140-6736(07)61306-3

[32] Hibbeln JR, Davis JM (2009) Considerations regarding neuropsychiatric nutritional requirements for intakes of omega-3 highly unsaturated fatty acids. Prostaglandins Leukot Essent Fatty Acids 81: 179–186. 19619995 10.1016/j.plefa.2009.06.005 PMC3182570

