# Supplementary material for: Docosahexaenoic Acid for Reading, Cognition and Behavior in Children Aged 7–9 Years: A Randomized, Controlled Trial (The DOLAB Study)
Source: PLoS One. 2012 Sep 6;7(9):e43909. doi: 10.1371/journal.pone.0043909 (PMC3435388; doi:10.1371/journal.pone.0043909)
Supplement: Protocol S1 — Study protocol. (DOC) [file pone.0043909.s001.doc]

**The DHA (docosahexaenoic acid) Oxford Learning and Behaviour (DOLAB) Study**

**PROTOCOL**

Centre for Evidence Based Intervention

University of Oxford

Version 4 *

13^th^ February 2009

**CONFIDENTIAL**

***Protocol amended prior to first randomised child**

# TABLE OF CONTENTS

[**TABLE OF CONTENTS i**](#__RefHeading___Toc325029611)

[PROTOCOL SUMMARY 1](#__RefHeading___Toc325029612)

[Research Questions 1](#__RefHeading___Toc325029613)

[BACKGROUND AND RATIONALE 3](#__RefHeading___Toc325029614)

[Omega-3 for child behaviour and learning 3](#__RefHeading___Toc325029615)

[Study Populations 3](#__RefHeading___Toc325029616)

[Outcome Measures 4](#__RefHeading___Toc325029617)

[Why sleep might be a mediator 5](#__RefHeading___Toc325029618)

[Objectives 5](#__RefHeading___Toc325029619)

[TRIAL DESIGN 6](#__RefHeading___Toc325029620)

[Design 6](#__RefHeading___Toc325029621)

[Primary Endpoint 6](#__RefHeading___Toc325029622)

[Secondary Endpoints 6](#__RefHeading___Toc325029623)

[STUDY POPULATION 6](#__RefHeading___Toc325029624)

[Selection of participants 6](#__RefHeading___Toc325029625)

[Inclusion criteria 6](#__RefHeading___Toc325029626)

[Exclusion criteria 7](#__RefHeading___Toc325029627)

[Selection of schools 7](#__RefHeading___Toc325029628)

[School Inclusion criteria 7](#__RefHeading___Toc325029629)

[School Exclusion criteria 7](#__RefHeading___Toc325029630)

[STAGE ONE: ENROLMENT 7](#__RefHeading___Toc325029631)

[Screening 7](#__RefHeading___Toc325029632)

[Enrolment/ Baseline Assessments 8](#__RefHeading___Toc325029633)

[Randomisation Process 8](#__RefHeading___Toc325029634)

[STAGE TWO: TRIAL TREATMENTS 8](#__RefHeading___Toc325029635)

[Active supplementation 8](#__RefHeading___Toc325029636)

[Placebo 8](#__RefHeading___Toc325029637)

[Supply and Labeling 9](#__RefHeading___Toc325029638)

[Unblinding of Individual Participants During The Trial 9](#__RefHeading___Toc325029639)

[Assessment of Compliance with Study Treatment/s 9](#__RefHeading___Toc325029640)

[Early withdrawal 9](#__RefHeading___Toc325029641)

[Participant Transfer and Withdrawal 9](#__RefHeading___Toc325029642)

[Participant Transfers 9](#__RefHeading___Toc325029643)

[Withdrawal from Trial Intervention 10](#__RefHeading___Toc325029644)

[Withdrawal from Trial Completely 10](#__RefHeading___Toc325029645)

[Loss to Follow-up 10](#__RefHeading___Toc325029646)

[ASSESSMENTS AND PROCEDURES (1) - Overview 10](#__RefHeading___Toc325029647)

[Study Schedule 10](#__RefHeading___Toc325029648)

[Screening 11](#__RefHeading___Toc325029649)

[Baseline 11](#__RefHeading___Toc325029650)

[Post-intervention (Stage Two participants only) 12](#__RefHeading___Toc325029651)

[ASSESSMENTS AND PROCEDURES (2) – Details of measures 12](#__RefHeading___Toc325029652)

[Screening 13](#__RefHeading___Toc325029653)

[Key Stage 1 Reading score 13](#__RefHeading___Toc325029654)

[Baseline and post-intervention 13](#__RefHeading___Toc325029655)

[Conners Teacher and Parent Ratings (CTRS-L and CPRS-L) 13](#__RefHeading___Toc325029656)

[British Ability Scale (BAS II) Word reading 13](#__RefHeading___Toc325029657)

[British Ability Scale (BAS II) Recall of Digits 13](#__RefHeading___Toc325029658)

[Fingerprick blood measures to assess fatty acid status and compliance 14](#__RefHeading___Toc325029659)

[Estimate of treatment group 14](#__RefHeading___Toc325029660)

[Side effects scale 14](#__RefHeading___Toc325029661)

[Child Sleep Habits Questionnaire (CSHQ) 14](#__RefHeading___Toc325029662)

[Sleep Diary (see Appendix A) 14](#__RefHeading___Toc325029663)

[Actigraphy 15](#__RefHeading___Toc325029664)

[STATISTICAL CONSIDERATIONS 15](#__RefHeading___Toc325029665)

[Method of Randomisation 15](#__RefHeading___Toc325029666)

[Outcome Measures 15](#__RefHeading___Toc325029667)

[Primary outcome 15](#__RefHeading___Toc325029668)

[Secondary outcome 16](#__RefHeading___Toc325029669)

[Stage One (Observational study) 16](#__RefHeading___Toc325029670)

[Sample Size 16](#__RefHeading___Toc325029671)

[Analysis Plan 16](#__RefHeading___Toc325029672)

[PHARMACOVIGILANCE 17](#__RefHeading___Toc325029673)

[Potential Risks and Benefits of Omega 3s 17](#__RefHeading___Toc325029674)

[Potential Risks 17](#__RefHeading___Toc325029675)

[Potential Benefits 17](#__RefHeading___Toc325029676)

[ETHICAL CONSIDERATIONS 17](#__RefHeading___Toc325029677)

[Ethical considerations 17](#__RefHeading___Toc325029678)

[Ethical Approval 18](#__RefHeading___Toc325029679)

[Informed Consent Process 18](#__RefHeading___Toc325029680)

[TRIAL MONITORING 18](#__RefHeading___Toc325029681)

[Clinical Site Monitoring 18](#__RefHeading___Toc325029682)

[Direct Access to Data 18](#__RefHeading___Toc325029683)

[Confidentiality 19](#__RefHeading___Toc325029684)

[Quality Assurance and Quality Control of Data 19](#__RefHeading___Toc325029685)

[Records Retention 19](#__RefHeading___Toc325029686)

[INDEMNITY 20](#__RefHeading___Toc325029687)

[FINANCIAL ARRANGEMENTS 20](#__RefHeading___Toc325029688)

[Payroll Staff - Roles and Responsibilities 20](#__RefHeading___Toc325029689)

[TRIAL REGISTRATION AND STEERING COMMITTEE 20](#__RefHeading___Toc325029690)

[The Trial Steering Committee will be consulted quarterly and its role will be to provide guidance and advice on the following topics: 21](#__RefHeading___Toc325029691)

[PUBLICATIONS 21](#__RefHeading___Toc325029692)

[APPENDIX A: Sleep Diary 22](#__RefHeading___Toc325029693)

[APPENDIX B: Project Approval Form 23](#__RefHeading___Toc325029694)

[REFERENCES 25](#__RefHeading___Toc325029695)

# PROTOCOL SUMMARY

**Title:** The DHA (docosahexaenoic acid) Oxford Learning and Behaviour (DOLAB) Study.

**Type of Study:** The study has two stages: Stage One is an

observational study and will be followed by Stage Two, a randomised controlled trial.

**Population:** Up to 600 children aged 7 years to 9 years from mainstream schools who are currently underperforming in reading (lowest tertile on nationally standardized achievement tests) will be included in Stage One of the study. 360 of these children will go on to be included in Stage Two, the randomised controlled trial.

**Number of Sites:** Up to fifty schools in Oxfordshire (and Buckinghamshire and Berkshire if required).

**Intervention:** The active compound (DHA, 600mg per day) and placebo (matching in package, taste and appearance) will be administered in an oral supplement.

**Study Duration and process:**

In Stage One of the study the parents/guardians of up to 600 children who are underperforming in reading (according to national tests or school records will be invited to consent to their child participating in a short school-based session, involving brief assessments of reading and working memory, an optional pinprick blood sample, and ratings from their teachers and parents. 360 of these children, whose assessment scores at Stage One indicate that they may benefit from this intervention, will be invited to join Stage Two, a randomised controlled trial. Baseline measures will be collected after which children will be randomly allocated to Omega-3 (DHA) or placebo conditions and followed up after 16 weeks. Recruitment will be carried out over two years. A ten percent sub-group of children in both groups will be randomly allocated to objective sleep monitoring.

## Research Questions

**Stage Two (RCT)**

**Primary: Can supplementation with Omega 3 (DHA) improve child behaviour and learning in underperforming children aged 7-9 years?** Outcomes assessed will include reading performance, working memory and ADHD-type symptoms (inattention, hyperactivity, impulsivity) as rated by teachers and parents.

**Secondary:**

**Might any treatment effects on behaviour or cognition be mediated by changes in child sleep?** (measured subjectively in all participants and objectively in a subset)

**Stage One (Observational Study)**

**Secondary: Do children’s levels of DHA or related fatty acids (**assessed objectively via a pinprick blood sample**) predict their learning, behaviour or sleep?**

**Are any observed changes in behaviour or cognitive performance associated with changes in blood fatty acid status?**

**Do sleep measures (and any changes in these) relate to objective measures of blood fatty acid status?**

# BACKGROUND AND RATIONALE

## Omega-3 for child behaviour and learning

Mounting evidence from human clinical trials shows that an increased dietary intake of the highly unsaturated Omega 3 fatty acids found in seafood (EPA and DHA) may have significant benefits for various aspects of mood, behaviour and learning (Freeman *et al* 2006). In children, four small controlled treatment trials have shown benefits for behaviour and/or learning from supplementation with these Omega 3 (Richardson and Puri 2002, Stevens *et al* 2003, Richardson and Montgomery 2005, Sinn & Bryan 2006), while two others have not (Voigt *et al* 2001, Hirayama *et al* 2004).

These studies have varied in their methodologies with respect to outcomes, populations studied and formulations used (see Richardson 2006), but the most consistently reported benefits have been improvements in attention and concentration and reductions in disruptive or antisocial behaviour. Highly significant improvements in both reading and spelling performance were also found in the one study that assessed these outcomes (Richardson and Montgomery 2005).

Child behaviour and learning problems have become a major problem in the UK and other developed countries. Conditions like attention-deficit hyperactivity disorder (ADHD) and reading disabilities (dyslexia) now appear to affect at least one in five school-age children, but many more show similar difficulties in milder form.These conditions have substantial costs not only for those directly affected, but for society as a whole (Burd *et al* 2003), and their consequences are lifelong.

Drug treatments such as methylphenidate are increasingly used to treat child behaviour problems, but these have undesirable side-effects. They also show only limited benefits for the core behavioural symptoms and none at all for learning.(NIH, 1998) Confirmation that dietary supplementation with omega-3 can significantly improve children’s behaviour and/or boost their performance in literacy and related cognitive skills would therefore have very important implications for policy and practice.

## Study Populations

To date, trials with children have focused on particular subgroups with specific developmental disorders of behaviour and/or learning such as ADHD, dyslexia or developmental coordination disorder (DCD).

- **The key question that has not yet been addressed is whether benefits reported for child behaviour and learning from omega-3 supplementation may extend to the general school population.**

The Oxford-Durham study (Richardson and Montgomery 2005) focused on children who met DSM-IV criteria for DCD, but the vast majority had never previously been formally diagnosed with this or any other behavioural or learning disorder. The positive results found suggest that supplementation with omega-3 may help many other underperforming children in mainstream schools.

Around 1/3 of the Oxford-Durham study sample were also found to meet standard criteria for dyslexia / specific reading difficulties, while 1/3 scored in the clinical range for ADHD-type symptoms. This was fully expected given the usual high comorbidity between these developmental conditions - and the study was specifically designed to include reading, spelling and ADHD-type symptoms as primary outcomes.

As noted above, behaviour and learning difficulties of these kinds affect at least 20% of the school population, although the boundaries with normal function are unclear, and in practice most children with these kinds of difficulties are never formally diagnosed.

- **This study will therefore involve children attending mainstream schools who are underperforming academically (according to standardized national attainment tests).**

**To date, published studies in this area have been too small or unrepresentative to detect significant differences in treatment response according to eit**her age or sex.

Behaviour and learning difficulties usually compound with age, so early intervention is always preferable, but our clinical and research experience indicates that 7-9 year olds are the age group most likely to show significant improvements in literacy skills following omega-3 supplementation (or other interventions). Standardised national assessments of performance in a range of key areas (including literacy) are carried out at ages 7, 11 and 14 on all UK children attending state schools,[[1]](#footnote-2) and results from these can be used to select children who are underperforming for their age. For these reasons, this study will involve children aged between 7 and 9 years.

Published trials in this area have included mainly boys, who are more likely than girls to show overt behavioural and/or learning difficulties. The male:female ratio was lowest in the Oxford-Durham study (at around 3:1), but many females in this study showed a very good response to supplementation, an impression supported by our clinical work and unpublished pilot data. This study will therefore focus simply on children who are underperforming for their age, although possible sex differences will be explored in the analyses.

## Outcome Measures

With respect to behaviour, clinical rating scales are rarely sensitive enough to detect treatment effects, and neither these nor various computer tests designed to assess attention or impulsivity have proved successful to date (Voigt *et al*, 2001; Stevens *et al* 2003; Hirayama*, et al.*, 2004). In this study, behaviour will be assessed by both teachers and parents, using well-validated questionnaire rating scales that are (a) psychometrically sound, (b) suitable for general population use and (c) likely to be sensitive to any treatment effects. The Conners’ Scales (Conners 1997) were originally developed to assess pharmacological treatments for child behaviour problems, and have shown benefits in three of the successful RCTs of omega-3 for child behaviour and learning (Richardson & Puri, 2002; Richardson & Montgomery, 2005; Sinn & Bryan 2006)

With respect to cognitive function, simple, transparent measures are again likely to be the most useful. The Oxford-Durham study showed clear benefits from fatty acid treatment for literacy skills (reading and spelling), and also on a simple, widely used test of working memory. Age-standardised tests of reading performance and working memory will therefore be used as primary outcomes in this study.

## Why sleep might be a mediator

Our own clinical and research work (jointly and independently) has long suggested that many of the benefits for mood, behaviour and cognition reported following omega-3 supplementation may in fact be mediated by improvements in sleep. ADHD and related child behaviour and learning difficulties are commonly associated with sleep problems (independently of medication effects), and these can exacerbate symptoms such as impulsivity or emotional lability as well as impairing attention, working memory and other aspects of cognition.

Many fatty acid derivatives play key roles in sleep initiation and maintenance (Yehuda 1998). Preliminary evidence from animal studies also suggests that fatty acid supplementation may help to reduce some of the anxiogenic effects of REM sleep deprivation (Yehuda 2007), and controlled studies of the effects of omega-3 on sleep in humans are now clearly warranted.

This study will therefore include simple subjective measures of sleep on all participants, as well as objective measures (using actigraphy) in a subset of children. If DHA supplementation could be shown to improve sleep parameters alone, this would be an extremely important finding with applicability to many other conditions. The ability to determine whether sleep may help to mediate any changes in child behaviour and cognition, and to explore associations with biochemical measures of fatty acid status, will also add considerably to the value of this study.

## Objectives

The primary objective of this trial is to determine whether DHA (in a daily dose of 600 mg.) will improve the behaviour and learning of normal children aged 7-9 years in mainstream state schools who are underperforming according to nationally standardized tests (≤ 33^rd^ centile in literacy skills) but who had no other significant learning difficulties according to their teachers.

Secondary objectives include determining (a) whether blood biochemical measures of fatty acid status are associated with behavioural and cognitive outcomes, and with sleep; and (b) whether DHA supplementation changes children’s sleep, and if so, whether this may mediate any changes in behaviour and learning.

# TRIAL DESIGN

## Design

Stage One is an observational study of up to 600 underperforming children aged 7-9 years from the general school population. 360 children, whose assessment scores at Stage One indicate that they may benefit from the study intervention, will enter a

randomised controlled trial of DHA vs placebo for 16 weeks (fixed dose, parallel group study).

## Primary Endpoint

1. To confirm or refute that supplementation with Omega-3 fatty acids (DHA, 600mg/day) for 16 weeks improves behaviour and learning in children aged 7 – 9 years from mainstream schools who are underperforming academically.

## Secondary Endpoints

1. To assess whether levels of DHA or related fatty acids (assessed objectively via a pinprick blood sample) predict their learning, behaviour or sleep.
2. To assess whether any changes in behaviour or cognitive performance are associated with changes in objective measures of blood fatty acid status.
3. To assess whether any treatment effects on behaviour or cognition are mediated by changes in child sleep (measured subjectively in all participants and objectively in a subset).
4. To explore associations between child sleep and blood fatty acid status (at each timepoint, and with respect to changes following treatment).

.

# STUDY POPULATION

## Selection of participants

### Inclusion criteria

1. Children aged 7 – 9 years from mainstream state schools who are underperforming in literacy skills according to nationally standardized assessments of scholastic achievement at age 7 years (Key Stage 1[[2]](#footnote-3)). To be eligible, children must score ≤ 33^rd^ centile for reading but be judged by their teachers to have no other significant learning difficulty.

**2**. English as a first language.

### Exclusion criteria

1. Major learning disabilities or medical disorders
2. Taking medications expected to affect behaviour and learning
3. Taking fish oils already, or eating fish > 2 x week

## Selection of schools

School selection will be critical to the efficient conduct of the study. Outline permission for the study has already been given by Senior Executives at Oxfordshire Education Authority, which has a more than sufficient number of primary schools, but if necessary, suitable schools from neighbouring counties of Berkshire and Buckinghamshire will also be considered.

### School Inclusion criteria

- Mainstream local authority primary schools in Oxfordshire, Berkshire and Buckinghamshire
- Educational authority ethics approval
- Agreement of head and participating teachers

### School Exclusion criteria

- Not meeting the inclusion criteria listed above

Subject to the above, each participating school will be carefully identified on the basis of:

- Having at least one teacher with a specific interest in, and responsibility for, supervising and managing suitable children.
- Showing enthusiasm to participate in the study.
- Ensuring that sufficient time, staff and adequate facilities are available for the trial.
- Providing information to all supporting staff members, parents and children involved with the trial.
- Acknowledging and agreeing to conform to the administrative and ethical requirements and responsibilities of the study, including signing-up to Good Clinical Practice and other regulatory documentation.

# STAGE ONE: ENROLMENT

## Screening

Screening based on the results of age 7+ Key Stage I assessments will be performed to assess a child’s possible eligibility for the study.

Parents/carers of eligible children will be provided with information on the study and invited to consent to their child’s participation.Fully informed written proxy consent (and assent) to participate in the study will be required before any baseline assessments are carried out.

## Enrolment/ Baseline Assessments

The following baseline measures will be obtained on participating children:

1. Teacher: Conners Teacher Ratings (CTRS-L) (Conners 1997)
2. Parent: Conners Parent Ratings (CPRS-L) (Conners 1997)

Demographics

Child Sleep Habits Questionnaire (CSHQ) (Owens 2000)

3. Child: Word reading and Recall of digits (British Ability Scale - BAS II) (Elliott, 1997)
Fingerprick blood measures to assess fatty acid status and compliance

Sleep measured objectively by actigraph on a 10% subset.

## Randomisation Process

1. Fully informed written proxy consent
2. Confirmation of eligibility, randomisation and issue of allocated treatment by Centre for Statistics in Medicine, University of Oxford (see later).

# STAGE TWO: TRIAL TREATMENTS

Stage Two of the study is a simple parallel group randomised double-blind controlled trial comparing the effects of active supplementation with Omega-3 (DHA) at a fixed dose (600mg DHA per day) versus taste and colour-matched placebo. DHA or placebo supplements will be supplied in numbered bottles to be delivered once daily to participating children over 16 weeks by teachers (Monday – Friday) and by parents/carers at weekends and during school holidays.

## Active supplementation

The active intervention will consist of 3 x 500 mg capsules per day, each capsule providing 200 mg of DHA as a triglyceride. The liquid fill contains DHASCO®-S oil, derived from the microalgae, *Schizochytrium sp.,* high-oleic sunflower oil, natural mixed tocopherols, ascorbyl palmitate, and rosemary extract (flavouring). The gelatin shell contains glycerin, water, and colouring (carmel, carmine, turmeric).[[3]](#footnote-4)

## Placebo

The placebo will consist of 3 x 500 mg capsules per day containing high-oleic sunflower oil. The dimensions, taste, appearance and colour will be identical to those of the DHA capsules. The shell of the capsule will be the same as the DHA capsule. The liquid fill contains high-oleic sunflower oil, natural mixed tocopherols, ascorbyl palmitat and rosemary extract (flavouring).

## Supply and Labeling

The active supplement and placebo will be supplied in 100 count bottles. Each participant will be required to consume 3 capsules daily (600 mg of DHA). Each bottle will be labeled with a 2-part label. The inner portion contains the protocol number, the participant random allocation number, a place to record the participant’s initials, storage instructions, sponsor and PI’s name and address and an Investigational Product Statement. The outer tear-off portion of the bottle label contains the protocol number, the participant random allocation number and a place to record the participant’s initials.

## Unblinding of Individual Participants During The Trial

Unblinding will be considered only when knowledge of the treatment assignment is deemed essential for the child’s care by their physician or a regulatory body. In general, unblinding of participants during the conduct of the clinical trial is not allowed unless there are compelling medical or safety reasons to do so. The decision to unblind a single case will only be made when knowledge of an individual’s allocated treatment is essential to:

- Enable treatment of severe adverse event/s, or
- Enable administration of another therapy that is contraindicated by the trial treatment.

Where possible, the agreement of the lead investigators (Dr Alex Richardson and/or Dr Paul Montgomery) will be sought before requests for individual unblinding are made.

## Assessment of Compliance with Study Treatment/s

At the end of each child’s participation in the study, a member of the research staff will collect all unused supplements allocated to that child, and a pill count will be conducted and recorded to determine the doses administered. All unused medication collected will be returned to the research centre where they will be retained for the duration of the trial; at the end of the trial they will be collected for destruction.

## Early withdrawal

If a patient wishes to withdraw from trial treatment, all unused medication will be collected and a pill count conducted and recorded. The unused medication will be returned and retained for the duration of the trial; at the end of the trial they will be collected for destruction.

## Participant Transfer and Withdrawal

In consenting to the trial, participants are consenting to trial treatment, follow-up and data collection. If voluntary withdrawal occurs, the parent/carer will be asked to allow continuation of scheduled evaluations and to complete an end-of-study evaluation. Follow-up of these participants will be continued through the trial.

### Participant Transfers

Where possible we will aim to screen for these in advance and exclude if the child is known to be moving. If any child does transfer we will attempt to follow him/her up at the new school.

### Withdrawal from Trial Intervention

Participants may be withdrawn from the intervention for any of the following reasons:

1. Parent/ carer of the child withdraws consent for treatment.
2. Unacceptable adverse effects.
3. Intercurrent illness preventing further participation.
4. Development of serious disease preventing further participation.
5. Any change in the participants’ condition that justifies the discontinuation of participation in the opinion of parent, teacher or clinician.

If a parent/carer wishes to withdraw a child from the intervention, schools will inform the project administrator who should document the reason. The project coordinator will attempt to contact the parent/carer, explain the importance of remaining in the trial and being followed-up, and request that data be collected as per the trial schedule. Generally, follow-up will continue unless the parent/carer explicitly also withdraws consent for follow-up (see following section).

### Withdrawal from Trial Completely

Parents/carers are free to withdraw consent at any time without providing a reason. Those who do this will have anonymised data collected up to the point of that withdrawal of consent included in the analyses. The child will no longer receive a daily dose of either active treatment or placebo. Data up to the time of withdrawal will be included in the analyses unless the parent/carer explicitly states that this is not their wish.

## Loss to Follow-up

If any of the trial patients are lost to follow up, contact will initially be attempted through the school and parent address, otherwise missing data will be imputed using median values by treatment group.

# ASSESSMENTS AND PROCEDURES (1) - Overview

Recruitment will be carried out over two years. There will be two assessment points for children in Stage One of the study: screening and baseline. Children in Stage Two of the study will be assessed at three points: screening, baseline and post-intervention.

This section will outline the overall study schedule, the measures that will be administered at each of the assessment points (and by whom), how they will be performed and what will be done with these data.

The following section will provide further details of each of the assessments used.

## Study Schedule

The study will run for 28 months: September 2008 to December 2010. Recruitment for the 16 week intervention will be carried out over two years commencing January 2009 and finishing in July 2010.

NB: The exact study schedule will be planned in detail with participating schools according to term length and any school activities which could interfere with testing sessions (such as examination, sporting or other activities).

Data analysis and write up will be conducted between July and December 2010.

## Screening

Children aged 7-9 years (inclusive) whose Key Stage 1 scores at age 7 years place them within the lowest third for literacy, but whose teachers judge them tohave no other significant learning difficulty.

Research staff will check with the school whether the child meets other eligibility criteria, i.e. has no major learning disabilities or medical disorders

If the child passes this initial school screening stage, teachers will be asked to confirm whether their reading is still a cause for concern. If it is not they will no longer be considered for inclusion in the study. Teachers will be asked to add the names of any additional children whose reading shows room for improvement.

Parents of potential participants will be contacted via a letter from their child’s school briefly describing the study. More detailed participant information will also be mailed with this letter along with a simple version for children, and the consent form and a freepost envelope addressed to their child’s school.

The invitation letter will ask parents to let the school know if they do not wish the school to give the researchers their contact details. Unless parents express a wish not to be contacted, this letter will be followed by a phone call from the researchers in which parents will be given the opportunity to ask questions and discuss the study. If necessary, arrangements will be made to either visit the parents at home or meet them at school.

Parents of children in Stage One of the study will be asked to provide assessments at baseline, and those in Stage Two will be asked to provide these same assessments again post-intervention. It will be made clear to all parents that if their child is chosen to take part in Stage Two of the study they will be asked to give their child supplements each day during weekends and school holidays.

## Baseline

Confirmation that signed consent forms are in the PIs possession is required before baseline visits to schools will be arranged. The following measures will be administered in the school by the research staff:

Teacher: Conners Teacher Ratings

Child: Reading test age

Working memory digit span test

Fingerprick blood measures to assess compliance

At this point assessment measures will be scored by researchers. Children who meet inclusion criteria for Stage Two will be identified and randomization will be performed by telephone.

The following measures will be collected from parents using Freepost mail services and chased up with telephone calls or visits as required.

Parent: Conners Parent Ratings

Health questionnaire

Demographics

Child Sleep Habits Questionnaire (CSHQ)

On a 10% block random selected subsample of Stage Two participants, we will collect sleep measures objectively by actigraphy. This will require a home visit before supplementation begins to provide the actigraph and explain how to use it, and another home visit to collect it 5 days later.

## Post-intervention (Stage Two participants only)

Teacher: Conners Teacher Ratings

Estimate of treatment group

Child: Reading test age

Working memory digit span test

Fingerprick blood measures to assess compliance

Estimate of treatment group

The following measures will be collected from parents using Freepost mail services and chased up with telephone calls or visits as required.

Parent: Conners Parent Ratings

Estimate of treatment group

Child Sleep Habits Questionnaire (CSHQ)

Barkley Side Effects Rating Scale (SERS) (Barkley, 1990)

On the same selected subsample, we will again collect sleep measures objectively by actigraphy and derive the CSDI from the sleep diary.

Participants in both stages of the study will be given a 3 month supply of DHA (Life’s DHA, provided by Martek Biosciences Corporation) as a token of thanks for their participation in the study.

In due course, the parents of all participating children will also receive details of the overall study results, as will all participating schools.

# ASSESSMENTS AND PROCEDURES (2) – Details of measures

## Screening

### Key Stage 1 Reading score

Key Stage tests are nationally standardized assessments taken by all UK children at ages 7, 11 and 14. They encompass five areas: speaking and listening, reading, writing, science and mathematics.

Reading scores from Key Stage 1 tests will be used to screen for children performing in the lowest third for their age against national norms at age 7. To be eligible for the study, children must be judged by teachers to be performing normally in at least one other domain.

## Baseline and post-intervention

### Conners Teacher and Parent Ratings (CTRS-L and CPRS-L)

These are standardized highly valid and reliable scales which measure child behaviour over several domains, yielding age-standardised scores for 7 different sub-scales (6 for teacher ratings) and 7 global scales.

These scales have shown significant improvements following fatty acid supplementation in several previous trials, and have been routinely used in medication trials for children with behaviour problems such as ADHD.

Teacher ratings will be the primary outcome measure in both Stage One and Stage Two as this is a school-based study, but every effort will be made to ensure that parents also complete ratings pre- and post-treatment. By taking measures from both parents and children at Stage Two we will be able to assess whether any changes in behaviour can be seen in different situations.

### British Ability Scale (BAS II) Word reading

This is an age standardized single word reading test from the British Ability Scales, normed on UK children It is administered to each child individually, taking only a few minutes to complete.

This kind of reading test was used successfully in the Oxford-Durham trial and was sensitive enough to show significant change over three months.

### British Ability Scale (BAS II) Recall of Digits

This is a widely accepted measure of ‘working memory’ from the British Ability Scales. Again it is age standardized and administered at the individual level.

In the Oxford-Durham study, active treatment was associated with highly significant improvements over placebo in working memory performance, as assessed using this kind of test (, although this was only a secondary outcome., . Working memory problems are common to a wide range of different conditions in which omega-3 supplementation has been found beneficial including dyslexia, dyspraxia, ADHD, depression and age-related cognitive decline. Improvements in working memory are also likely to benefit many different aspects of cognitive performance in the general population.

### Fingerprick blood measures to assess fatty acid status and compliance

A fingerprick blood test will be performed at baseline for children in Stages One and Two andat the end of the study for children in Stage Two, in order toassess levels of fatty acid including docosahexaenoic acid (DHA), arachidonic acid (ARA), and eicosapentaenoic acid (EPA). Analysis of the fatty acids levels will be performed at Martek Biosciences Corporation, and the investigators will remain blinded to these until treatment codes are unlocked.

The fatty acid data will be used to verify compliance and to determine the relationship between levels of fatty acids in the blood and specific endpoints.

This blood test will be optional and will appear as a separate item on the consent form, but every effort will be made to maximize participation.

### Estimate of treatment group

The value of this measure is that it tests that the participants have remained blind to their group allocation.

### Side effects scale

Side effects will be measured post-intervention in all children participating in Stage Two using the Barkley Side Effects Rating Scale (SERS) (Barkley 1990). This scale was designed for use in studies of stimulant medication for ADHD-type symptoms. Based on our own and others’ previous studies, negative side effects are not expected from supplementation with DHA, but we are conscious that few studies specifically ask about these effects, so the use of this scale will redress that omission.

### Child Sleep Habits Questionnaire (CSHQ)

The Children’s Sleep Habits Questionnaire (CSHQ) will be completed at baseline by all children participating in Stage One and at post-intervention by all children participating in Stage Two.

This questionnaire, for children aged 4 to 12 years, is designed to screen for the most common sleep problems in that age group. The CSHQ focuses on sleep disorders common to this age group in three domains: Dyssomnias (difficulty getting to sleep or staying asleep, Parasomnias (sleepwalking/talking, night terrors, bedwetting, restless leg syndrome, etc), and Sleep-Disordered Breathing (Owens 2000).

In a 10% subset of children in Stage Two of the study, the duration of night-time sleep and sleep latency will be recorded by additional subjective (sleep diary) and objective means (actigraphy).

### Sleep Diary (see Appendix A)

Parents of the children whose sleep will be objectively measured will be asked to document on the form their child’s bed time and the time that they arise, indicating any intervals where the actigraph may be removed, and periods of sleep. The diary therefore allows parental perception of their child’s sleep to be documented and can be crosschecked with the actigraph data.

### Actigraphy

Actigraphy is the use of accelerometers to measure human movement. This technique has been used world-wide in a variety of research and clinical situations, and provides simple, objective measures of sleep efficacy without the need for intrusive electrode monitoring. The actigraph is worn on the wrist and the movement of the wrist is monitored continuously whilst it is being worn. The actigraph is very light-weight and can be used on individuals of all ages for long periods of time. Wrist movement data is processed within the unit and by subsequent software programs to give an indication of general activity levels.

At the pre-treatment baseline, parents will be asked to ensure that their child wears the actigraph at night for 5 nights. Post-intervention, they will be asked to do the same for a further 5 nights. Using the actigraph Sleep Analysis software, the following information will be recorded for each night of the sleep study period:

- total sleep time
- total wake time
- sleep onset latency
- number of awakenings

Data for each of these variables will be averaged over the 5-night period.

# STATISTICAL CONSIDERATIONS

## Method of Randomisation

Randomisation of children in stage two will be performed centrally at the Centre for Statistics in Medicine in Oxford (CSM) using dedicated computer software. Following recruitment and consent, participants’ details will be faxed to the CSM. The randomisation programme will include a minimisation algorithm to ensure balanced allocation of participants across the treatment groups for school (to allow for any sociodemographic/school differences) and sex of the child (an important prognostic factor). The first 36 patients (10%) will be allocated randomly without the minimisation factors to avoid predictability. The minimisation algorithm will be applied with an allocation ratio that is not fully deterministic. Fifteen percent of the sample will be randomly selected to use the objective sleep monitors (allowing for some refusals). The statistician carrying out the randomisation will have no direct contact with participants so allocation will be totally independent of patient recruitment.

## Outcome Measures

Stage Two (RCT)

### Primary outcome

- Can supplementation with Omega 3 (DHA) improve child behaviour and learning in underachieving children aged 7-9 years? Outcomes assessed will include reading performance, working memory and ADHD-type symptoms (inattention, hyperactivity, impulsivity) as rated by teachers and parents.

### Secondary outcome

- Might any treatment effects on behaviour or cognition be mediated by changes in child sleep? (measured subjectively in all participants and objectively in a subset)

### Stage One (Observational study)

Secondary outcomes

- Do children’s levels of DHA or related fatty acids (assess objectively via a pinprick blood sample) predict their learning, behaviour or sleep?
- Are any changes in behaviour or cognitive performance associated with changes in blood fatty acid status?
- Do sleep measures (and any changes in these) relate to objective measures of blood fatty acid status?

## Sample Size

At Stage One, our previous studies indicate that a sample of 600 participants will be needed to identify an adequate number of eligible participants* for the intervention study, for which power calculations have been carried out (see below). This number of Stage One participants will also provide sufficient variance on all secondary outcome measures to ensure valid correlational analyses between these.

*Recruits to Stage Two must show ‘room for improvement’ on the main primary outcome (i.e. scoring within the lowest tertile on age-standardised measures of reading).

Following the only previous randomized controlled study to assess reading ability, power calculations were performed to estimate likely group sizes (Richardson & Montgomery, 2005)). These indicated that with significance set at 5% and power at 90%, groups of 180 (i.e. a total n of 360) would be required, based on our standardized reading change. Other outcomes would have required smaller sample sizes.

## Analysis Plan

The study will be analysed and reported following the ‘CONSORT’ guidelines (Consolidated Standard of Reporting Trials) (Moher, 2001).

On completion of the study post-intervention change score data from all children participating in Stage Two will be analysed on an intention to treat basis. Since the original protocol required that children be below the 20th centile on a standardized word reading test, ITT analysis of post-intervention change score data on these children will also be carried out. To further assess the importance of severity of reading impairment at baseline, similar analyses will be performed on children whose reading was assessed ≤ 10% level.

Missing data will be handled by imputing median values for missing data by treatment group.

# PHARMACOVIGILANCE

This trial will be conducted in accordance with the terms and conditions outlined in the Medicines for Human Use (Clinical Trials) Regulations 2004 (SI 2004/1031).

## Potential Risks and Benefits of Omega 3s

### Potential Risks

None are known. According to the US FDA, 3g per day of EPA + DHA is generally regarded as safe (GRAS). This study will use only 600mg.

Clinical studies of omega-3 supplementation in humans (adult and child volunteers and patients of both sexes and all ages) have not shown any consistent or serious short or long-term adverse side-effects.

Martek’s DHA is already is widely used in superior infant formulae. The FDA considers both DHASCO®-S and high-oleic sunflower oil to be “highly refined oils” that are not associated with allergic reactions.[[4]](#footnote-5)

### Potential Benefits

Clearly, benefits can never be guaranteed for individual participants in any research trial, but average intakes of DHA in the UK and other developed countries are widely acknowledged to be suboptimal for general health.(Hibbeln *et al* 2006) Adequate supplies of DHA are essential for optimal development and functioning of the brain and nervous system (particularly for vision), and for cardiovascular and immune health.

As noted above, preliminary RCT evidence from studies of healthy children has already shown improvements in attention, concentration and behaviour, and benefits for literacy skills. The aim of this study is to confirm and extend these findings.

# ETHICAL CONSIDERATIONS

## Ethical considerations

The study will abide by the principles of the World Medical Association Declaration of Helsinki (1964) and the subsequent revisions; Tokyo (1975), Venice (1983), Hong Kong (1989) and South Africa (1996). We consider the specific ethical issues relating to participation in this trial to be:

- Taking a placebo (dummy) treatment or the active (Omega-3) treatment.

There is no single, 'gold standard' treatment that is used to combat scholastic underachievement and/or behaviour problems in children. Those drugs that are sometimes prescribed for behaviour problems associated with ADHD have obvious and unpleasant side-effects that would make blinding difficult in any case, but more fundamentally, they are not an appropriate intervention for normal children who are simply underperforming at school. Placebo is therefore an appropriate comparator because (a) there is no standard treatment and (b) double-blinding enables the true treatment effect of DHA to be investigated.

- Case Report Forms and Assessments in English only

To provide appropriate translations of all trial documentation and assessments would require checks and validations of language, as well as a comprehensive review of cultural and linguistic influences on the study measures. The latter include age-standardised tests of literacy skills, which are likely to be affected by the primary language spoken. We unfortunately do not have the resources to fulfil these standards adequately and therefore must limit trial entry to children with English as a first language.

## Ethical Approval

The trial protocol is in preparation for submission to the Integrated Research Application System and to the Oxfordshire County Council Education Department (Appendix B).

## Informed Consent Process

Informed consent is a process initiated prior to an individual agreeing to participate in a trial and continues throughout the individual’s participation. Informed consent is required for all patients participating in coordinated trials. In obtaining and documenting informed consent, the investigators will comply with applicable regulatory requirements and adhere to the ethical principles that have their origin in the Declaration of Helsinki.

# TRIAL MONITORING

## Clinical Site Monitoring

### Direct Access to Data

Site monitoring may be deemed to be necessary as a result of central data checks. In order to perform their role effectively, the trial coordinator and others involved in Quality Assurance and Inspection may need direct access to primary data, eg school records, relevant medical reports etc. Since this affects the patient’s confidentiality, this fact is included on the Patient Information Sheet and Informed Consent Form.

### Confidentiality

Individual participant medical information obtained as a result of this study is considered confidential, and disclosure to third parties is prohibited with the exceptions noted below.

Electronic and paper record forms will be labelled with patient initials and unique trial registration and/or randomisation number. Verification of appropriate informed consent will be enabled by the provision of copies of participants’ signed informed consent/assent forms being supplied to the schools.

The Centre for Evidence Based Intervention (CEBI) will preserve the confidentiality of participants taking part in the study and The University of Oxford is registered under the Data Protection Act.

### Quality Assurance and Quality Control of Data

QA includes all the planned and systematic actions established to ensure the trial is performed and data generated, documented/recorded and reported in compliance with applicable regulatory requirements.

QC includes the operational techniques and activities done within the QA system to verify that the requirements for quality of the trial-related activities are fulfilled.

This trial will undergo a risk assessment, the outcome of which is likely to indicate that it is a low risk trial. As such, site visits will be conducted and source data verification performed if indicated to be required as a result of central monitoring processes. To this end:

- The Principal Investigators, the Trial Coordinator and other staff will attend the launch meetings for key staff from participating schools, which will incorporate elements of trial-specific training necessary to fulfill protocol requirements.
- The Trial Coordinator will verify that appropriate approvals are in place prior to initiation of a school, and to check that the relevant personnel have attended trial-specific training.
- The Trial Coordinator will check safety and compliance reporting rates between centres.
- The Trial Coordinator will monitor screening, recruitment and drop-out rates between schools.
- The Trial Coordinator will conduct data entry consistency checks and follow-up data queries.
- Independent oversight of the trial will be provided by the Trial Steering Committee, to which Drs Montgomery and Richardson will play a reporting role.

## Records Retention

The CEBI undertake to store originally completed record forms and separate copies of the above documents for up to a maximum of 3 years or until the University informs the investigators that the documents are no longer to be retained.

Arrangements will be made to ensure the continued storage of the documents, even if the investigators, for example, leave the University or retire before the end of required storage period. Delegation will be documented in writing, and the investigators will archive all relevant source documents so that the trial data can be compared against source data after completion of the trial (e.g. in case of inspection from authorities).

# INDEMNITY

Indemnity for this trial will be provided by the University of Oxford.

# FINANCIAL ARRANGEMENTS

These have been negotiated between the Research Services Office at Oxford University and Martek Biosciences

### Payroll Staff - Roles and Responsibilities

**Drs. Montgomery and Richardson** will be the joint ***primary investigators*** taking overall responsibility for the research, including the trial design; directing, managing and supervising the implementation of the study; data analyses; and reporting and disseminating of the findings. They will ensure that all phases of research adhere to the highest scientific, ethical and other professional standards.

**Ms. Burton** will act as the full-time ***Trial Coordinator***. She is a highly experienced member of the research staff at the Centre for Evidence-Based Intervention who has run many randomised trials with children. Her role will involve liaising with schools and Local Education Authority staff to set up efficient systems for screening, recruitment, administration of treatments and data collection. She will be responsible for the day to day running of the study and data management, including supervising the data collection assistants throughout the course of the study. She will also work closely with Drs Montgomery and Richardson, assisting with the preparation of the final study protocol and research ethics applications, and also with the writing up of research publications.

***Richard Sewell will be Trial Administrator*** full-time on the study, and will be responsible for all routine administration, including distribution and collection of information and consent forms, questionnaire measures, and actigraphs; and ensuring that supplies of intervention and placebo are delivered to and collected from schools according to protocol. The Administrator will also be responsible for data entry and data cleaning, as well as acting as a central point of contact between schools and researchers throughout the course of the study.

***Data Collection Assistants*** (part-time, session-based) will visit schools under Ms Burton and Mr Sewell’s direction and supervision, as well as carrying out some home visits (for sleep measures and/or collecting data needed from parents’ questionnaires). Their role will centre on administration of questionnaire and other measures at screening, baseline and post-intervention, but will also involve ‘troubleshooting’, i.e. chasing up of participants when necessary (e.g. if scheduled school appointments cannot be met)

# TRIAL REGISTRATION AND STEERING COMMITTEE

This trial will be registered in advance with the Current Controlled Trials (<http://www.controlled-trials.com/>) and ClinicalTrials.gov <http://clinicaltrials.gov/>).

The Trial Steering Committee will consist of:

- Professor John Stein, Professor of Neurophysiology, University of Oxford
- Professor Frances Gardner, Professor of Child and Family Psychology, University of Oxford
- Professor Crispin Jenkinson, Director, Health Services Research Unit, University of Oxford
- Dr Karen Smith, Statistician, Centre for Statistics in Medicine, University of Oxford
- Dr Alexandra Richardson, University of Oxford
- Dr Paul Montgomery, University of Oxford

# The Trial Steering Committee will be consulted quarterly and its role will be to provide guidance and advice on the following topics:

- Recruitment
- Randomisation
- Trial progress
- Protocol amendments
- Ethics
- Budget
- Adverse events and serious adverse events

# PUBLICATIONS

It is anticipated that at least three strong academic papers will be forthcoming from this study: one on the primary outcomes (to address whether DHA improves child behaviour and learning); a second on the objective blood biochemical measures and their correlations with the outcome measures; and a third looking at the role of sleep in relation to DHA and its effects on behaviour and learning. The data generated should also provide material for further academic publications in addition to these major ones.

We will also produce briefer papers for the ‘trade press’ as well as website material for posting on the University of Oxford Centre for Evidence Based Intervention website, and any such materials will be available for Martek Biosciences Corporation to use as they see fit.

Dr Paul Montgomery

Dr Alexandra Richardson

# APPENDIX A: Sleep Diary

| Date |  |  |
| --- | --- | --- |
| Time woke/woken |  |  |
| Time got up |  |  |
| Any problems on waking? Please describe. |  |  |
| Time and length of any daytime naps. What were they doing just before they napped (eg. at school, in the car, watching TV etc.) |  |  |
| Times during the day when your child seemed sleepy (although didn't nap). What were they doing at this time (eg. at school, in the car, watching TV etc.) |  |  |
| Time to bed |  |  |
| Time to sleep |  |  |
| Any problems going to bed/getting off to sleep. Please describe including what you did, what your child did and how they eventually fell asleep. Please indicate if your child seemed upset during any wakes or was simply awake yet content. |  |  |
| Time and length of any wakes during the night. Please describe why they woke (if known), what they did, what you did and how they eventually fell asleep. |  |  |
| Times of breakfast (B), lunch (L) and dinner (D) | B  L  D | B  L  D |
| Anything else of importance (day or night) |  |  |

# APPENDIX B: Project Approval Form

| **PROJECT title –** The effects of Omega 3 (DHA) on Child Behaviour and Learning | |
| --- | --- |
| **Proposer:**   1. Peter Wild, CYP&F   **2.** Dr . Paul Montgomery – University  of Oxford | **Date of proposal:**  April 2008 |
| **Job title of proposer:**   1. Assistant Head of Service YP&AE   **2 .** University Lecturer in Evidence Based Intervention | |
| **Overall purpose of project**:  Part of developing an evidence-based approach to meeting CY&F Outcomes in partnership with a University research team.  To emphasise cross cutting relationship between healthy lifestyles and good behaviour to optimise learning | |
| **Is the project linked to particular CYPP priorities**?  Annex 3  101/01 increase no. of healthy schools  301/03 raising standards  302/03 increasing enjoyment of education  303/05 improving performance at Key Stage 2 English  304/01 improved personal development and well being  402/01 improvement of behaviour /reduction of exclusions  possibly 402/04 reducing young entrants into criminal justice system  **How will it improve outcomes for CYP?**  Improved physical, emotional and mental health  Improved healthier lifestyles  Improved capacity to learn in core subjects  Improved behaviour  Better awareness of diet passed on to parents  **What level of impact is it designed to have?**  The project will provide a clear evidence base for the role of dietary supplements in enhancing pupil performance. This could lead to a more systematic approach to raising achievement and improving behaviour in mainstream school s by early intervention.  **If not linked to specific priorities, how more generally will the project enhance directorate capability and effectiveness? What problems is the project designed to overcome?**  Mitigate the effects of poor dietary habits and positively affect the well being and learning capabilities of a large cohort of under- performing children. | |
| **Timescales:** Planning phase between now and September 2008.  Project active between September 08 and April 2010  Research report available in 2010/2011 | |
| **Organisational impact:**  Research question:  Can supplementation with Omega 3 (DHA) improve child behavior and learning in underachieving children aged 7-9 years?  Evidence is building that Omega 3s have a significant effect on mood, behaviour and learning. What is not known, is whether there may be a benefit for children who are attending mainstream schools, have no diagnosis for any medical problem, but who are underachieving academically. Such children commonly have behavioural problems as well.  This project will provide all the necessary resources , however, CYP&F will need to support the study by way of helping the researchers engage with children and families at the various schools. Teachers will be asked to provide ratings of behavior of the children who are recruited into the study using a short well validated research measure. The dietary supplement will also be given to the children at school. | |
| **Financial implications:**  **Research costs met by research team :** Drs. Montgomery and Richardson are applying for full research funding for this 28 month study. It is not anticipated that taking part in it will involve any financial implications either for the Education Authority or the individual schools taking part.  Peripheral costs for organisational detail could be met from children’s services grant – see Peter Wild | |
| **View of the Head of Service:** | |
| **Comments of DLT** | |

# REFERENCES

Barkley RA (1990). Attention-Deficit Hyperactivity Disorder: A Handbook for Diagnosis and Treatment. New York, NY: Guilford Press

Burd L, Klug MG, Coumbe MJ. & Kerbeshian J (2003). Children and adolescents with attention deficit-hyperactivity disorder: 1. Prevalence and cost of care. Journal of Child Neurology, 18, 555-561.

Connors CK (1997). Conners’ Parenting Rating Scales – Revised. Technical manual. New York: Multi-Health Systems Inc.

Elliot, C., Smith, P. & McCulloch, K. (1996) British Ability Scales: Second Edition (BAS II). Windsor, UK: NFER-Nelson.

FDA (2005). The Threshold Working Group of the FDA. Approaches to Establish Thresholds for Major Food Allergens and for Gluten in Food. U.S. Food and Drug Administration, June 2005. Available at <http://www.cfsan.fda.gov/~dms/alrgn.html>

Freeman MP, Hibbeln JR, Wisner KL, Davis JM, Mischoulon D, Peet M, Keck PE Jr, Marangell LB, Richardson AJ, Lake J, Stoll AL (2006). Omega-3 fatty acids: evidence basis for treatment and future research in psychiatry. Journal of Clinical Psychiatry, 67(12): 1954-67.

Hirayama, S., Hamazaki, T. & Terasawa, K. (2004). Effect of docosahexaenoic acid-containing food administration on symptoms of attention-deficit/hyperactivity disorder - a placebo-controlled double-blind study. European Journal of Clinical Nutrition, 58, 467-473.

Hibbeln JR, Nieminen LRG, Blasbalg TL, Riggs JA, Lands WEM (2006). Healthy intakes of n-3 and n-6 fatty acids: estimations considering worldwide diversity. American Journal of Clinical Nutrition, **83**(suppl) 1483S-93S

Moher D, Schulz KF & Altman D (2001). The CONSORT statement: revised recommendations for improving the quality of reports of parallel-group randomised trials. Lancet, 357, 1191-1194.

Montgomery P, Stores G & Wiggs L (2004). The relative efficacy of two brief treatments for sleep problems in young learning disabled (mentally retarded) children: a randomised controlled trial. Archives of Disease in Childhood, 89 (2) 125-130.

NIH (1998). NIH Consensus Statement: Diagnosis and Treatment of Attention Deficit Hyperactivity Disorder (ADHD). Bethesda: National Institutes of Health.

Owens, J. A., Spirito, A., & McGuinn, M. (2000). The Children’s Sleep Habits Questionnaire. (CSHQ): Psychometric properties of a survey instrument for school-aged children. *Sleep, 23*(8), 1-9.

Richman N & Graham PJ (1971). A behaviour screening questionnaire for use with three year old children. Journal of Child Psychology and Psychiatry, 12, 5.33.

Richardson AJ & Montgomery P (2005). The Oxford-Durham study: a randomized controlled trial of dietary supplementation with fatty acids in children with developmental coordination disorder. Pediatrics 115 (5) 1360-1366.

Richardson AJ (2006). Omega-3 fatty acids in ADHD and related neurodevelopmental disorders. Int Rev Psychiatry, 18(2):155-72.

Richardson AJ, Puri BK (2002). A randomized double-blind, placebo-controlled study of the effects of supplementation with highly unsaturated fatty acids on ADHD-related symptoms in children with specific learning difficulties. Prog Neuropsychopharm Biol Psychiat, 26(2) 233-239.

Sinn N, Bryan J (2007). Effect of supplementation with polyunsaturated fatty acids and micronutrients on learning and behavior problems associated with child ADHD Journal of Developmental & Behavioural Pediatrics 28 82-91

Stevens L, Zhang W, Peck L, Kuczek T, Grevstat N, Mahon A, Zentall SS, Arnold LE & Burgess JR (2003). EFA supplementation in children with inattention, hyperactivity and other disruptive behaviours. Lipids, 38, 1007-1021.

Voigt RG, Llorente AM, Jensen CL, Fraley JK, Berretta MC & Heird WC (2001). A randomized, double-blind, placebo-controlled trial of docosahexaenoic acid supplementation in children with attention-deficit/hyperactivity disorder. Journal of Pediatrics, 139, 189-196.

Wechsler D (1993). Wechsler Objective Reading Dimensions (WORD): London, United Kingdon. The Psychological Corporation.

Wechsler D. (1991). Wechsler Intelligence Scale for Children-111. San Antonio, TX: The Psychological Corporation

Yehuda S, Rabinovitz S, Mostofsky DI (1998) Essential fatty acids and sleep: mini-review and hypothesis. Med Hypotheses. 50(2):139-45

Yehuda S, Rabinovitz S, Carasso RL, Mostofsky DI. (2007) Pretreatment with a mixture of essential fatty acids protects rats from anxiogenic effects of REM deprivation. Nutr Neurosci, 10(5-6):269-71.

1. These are known as ‘Key Stage’ assessments, and cover five major domains: reading, writing, speaking & listening, maths and science [↑](#footnote-ref-2)
2. Key Stage 1 assessments are carried out for all children attending UK state schools at the end of the school year in which they reach 7 years of age. The tests are nationally standardised, and cover five main areas: reading, writing, speaking and listening, maths, and science. [↑](#footnote-ref-3)
3. The oil encapsulation is performed by a validated Good Manufacturing Processing facility. The gelatin used for encapsulation protects the oil from oxidation and results in a capsule shelf life of two years or more when stored at room temperature. [↑](#footnote-ref-4)
4. In general, edible oils can be derived from major food allergens such as soybeans and peanuts, and may contain variable levels of protein. “The consumption of highly refined oils derived from major food allergens by individuals who are allergic to the source food does not appear to be associated with allergic reactions.” (The Threshold Working Group of the FDA, 2005) [↑](#footnote-ref-5)
